# Distribution, Function, and Evolution of a Gene Essential for Trichothecene Toxin Biosynthesis in *Trichoderma*

**DOI:** 10.3389/fmicb.2021.791641

**Published:** 2021-12-02

**Authors:** Santiago Gutiérrez, Susan P. McCormick, Rosa E. Cardoza, Hye-Seon Kim, Laura Lindo Yugueros, Martha Marie Vaughan, Guzmán Carro-Huerga, Mark Busman, Luis E. Sáenz de Miera, Walter M. Jaklitsch, Wen-Ying Zhuang, Chao Wang, Pedro A. Casquero, Robert Henry Proctor

**Affiliations:** ^1^University Group for Research in Engineering and Sustainable Agriculture (GUIIAS), Area of Microbiology, University of León, Ponferrada, Spain; ^2^USDA, Agricultural Research Service, National Center for Agricultural Utilization Research, Mycotoxin Prevention and Applied Microbiology Research Unit, Peoria, IL, United States; ^3^University Group for Research in Engineering and Sustainable Agriculture (GUIIAS), Area of Plant Production, University of León, León, Spain; ^4^Department of Molecular Biology, Area of Genetics, University of León, León, Spain; ^5^Division of Systematic and Evolutionary Botany, Department of Botany and Biodiversity Research, University of Vienna, Vienna, Austria; ^6^State Key Laboratory of Mycology, Institute of Microbiology, Chinese Academy of Sciences, Beijing, China

**Keywords:** *Trichoderma*, trichothecenes, *tri5* gene, positive selection, gene deletion and complementation, antifungal activity, genomics, phylogeny

## Abstract

Trichothecenes are terpenoid toxins produced by species in 10 fungal genera, including species of *Trichoderma*. The trichothecene biosynthetic gene (*tri*) cluster typically includes the *tri5* gene, which encodes a terpene synthase that catalyzes formation of trichodiene, the parent compound of all trichothecenes. The two *Trichoderma* species, *Trichoderma arundinaceum* and *T. brevicompactum*, that have been examined are unique in that *tri5* is located outside the *tri* cluster in a genomic region that does not include other known *tri* genes. In the current study, analysis of 35 species representing a wide range of the phylogenetic diversity of *Trichoderma* revealed that 22 species had *tri5*, but only 13 species had both *tri5* and the *tri* cluster. *tri5* was not located in the cluster in any species. Using complementation analysis of a *T. arundinaceum tri5* deletion mutant, we demonstrated that some *tri5* homologs from species that lack a *tri* cluster are functional, but others are not. Phylogenetic analyses suggest that *Trichoderma tri5* was under positive selection following its divergence from homologs in other fungi but before *Trichoderma* species began diverging from one another. We propose two models to explain these diverse observations. One model proposes that the location of *tri5* outside the *tri* cluster resulted from loss of *tri5* from the cluster in an ancestral species followed by reacquisition *via* horizontal transfer. The other model proposes that in species that have a functional *tri5* but lack the *tri* cluster, trichodiene production provides a competitive advantage.

## Introduction

Trichothecenes are toxins produced by a wide range of fungal species from three classes of the phylum Ascomycota (Proctor et al., 2020). However, most known trichothecene-producing fungi are members of class Sordariomycetes, order Hypocreales. These fungi include species of *Fusarium*, *Myrothecium*, *Stachybotrys*, and *Trichoderma*. Trichothecenes are of concern because of their toxicity and frequent occurrence in food and feed crops pose health risks to humans and domesticated animals (Desjardins, 2006). In addition, trichothecenes can serve as virulence factors in some crop diseases caused by *Fusarium* species (Desjardins et al., 1996). Finally, trichothecenes have been implicated in human diseases related to fungal contamination in damp buildings (Straus, 2009).

Over 200 trichothecene analogs have been reported (Proctor et al., 2018, 2020). All the analogs share a core chemical structure, 12,13-epoxytrichothec-9-ene (EPT) and differ from one another in the types and patterns of substitutions at various positions of EPT. Trichothecenes have been classified as macrocyclic or simple, based on the presence and absence of a macrolide ring formed by a 12 or 14-atom chain esterified to EPT *via* hydroxyl groups at carbon atoms 4 and 15. Macrocyclic trichothecenes have this macrolide ring, whereas simple trichothecenes do not (Proctor et al., 2018).

The genus *Trichoderma* includes fungi that can colonize a wide variety of substrates under diverse environmental conditions. These fungi can survive as saprobes on plant debris and, in some cases, can colonize living plant tissues (Morán-Díez et al., 2009). The ability of *Trichoderma* species to survive and compete in such diverse habitats has been attributed in part to their collective ability to produce diverse secondary metabolites, including antimicrobial compounds that can enhance their antagonistic activity (Sivasithamparam and Ghisalberti, 1998). Since 2005, production of at least eight trichothecene analogs has been reported in multiple *Trichoderma* species, although not all species were reported to produce all analogs (Lee et al., 2005; Nielsen et al., 2005; Degenkolb et al., 2008; Cardoza et al., 2011; Sun et al., 2016; Ryu et al., 2017; Chen et al., 2018). This production is intriguing, because some *Trichoderma* species are biological control agents for crop diseases caused by other fungi (Harman, 2000; Monte, 2001; Howell, 2003; Harman et al., 2004). The biocontrol activity of some trichothecene-producing *Trichoderma* species may result from induction of plant defense responses in addition to antifungal activity of certain trichothecene analogs against pathogenic fungi (Malmierca et al., 2013; Gutiérrez et al., 2020). In addition, a trichothecene analog produced by *T. albolutescens* was reported to have antiviral activity that contributed to protection against the Pepper Mottle Virus (Ryu et al., 2017).

In trichothecene-producing fungi that have been examined, the *tri5* gene encodes the enzyme trichodiene synthase, which catalyzes the cyclization of farnesyl diphosphate to trichodiene, the first committed intermediate in trichothecene biosynthesis. In most trichothecene-producing fungi, *tri5* is located in a trichothecene biosynthetic (*tri*) gene cluster (Lindo et al., 2018; Proctor et al., 2018). However, in *Trichoderma arundinaceum* and *T. brevicompactum*, the only two *Trichoderma* species whose *tri* clusters have been examined, *tri5* is located outside the cluster in a region that does not include other known *tri* genes (Cardoza et al., 2011; Lindo et al., 2018; Proctor et al., 2018).

The increasing number of reports on trichothecene production in *Trichoderma* species suggests that production could occur widely in the genus. Therefore, we used genome sequence, biochemical, molecular genetics, and phylogenetic analyses to further characterize trichothecene production in a collection of 35 species that represent a wide range of phylogenetic diversity that exists within *Trichoderma*. We focused on *tri5* because of its essential role in trichothecene biosynthesis combined with the uniqueness of its location outside the *tri* cluster in *T. arundinaceum*, *T. brevicompactum* and potentially other *Trichoderma* species (Cardoza et al., 2011; Proctor et al., 2020). Our results indicate that *tri5* is distributed widely but not universally in *Trichoderma*, while the *tri* cluster and trichothecene production are less widely distributed. Together, results from diverse analyses suggest models that can explain the location of *tri5* outside the *tri* cluster and why some *Trichoderma* species have retained a functional *tri5* in the absence of the cluster.

## Materials and Methods

### Nomenclature

Different multispecies lineages within *Trichoderma* have been designated using the terms “clade,” “section,” and “core group.” Most of the lineages have been assigned names: e.g., *Brevicompactum Clade*, *Section Trichoderma*, *Pachybasium* Core Group (Kubicek et al., 2019). However, all these subgeneric groups are phylogenetically distinct lineages within *Trichoderma*. For the purposes of this study, therefore, we have used a common term, lineage, to refer to multispecies subgeneric groups within *Trichoderma*. In addition, multiple formats of genetic nomenclature have been used for trichothecene biosynthetic and housekeeping genes from various fungi. For consistency within the current study, we use *Trichoderma* genetic nomenclature for all fungal species (e.g., *tri5* indicates a wild-type gene, and TRI5 indicates a protein).

### Strains Used and Growth Conditions

For this study, we selected 35 *Trichoderma* species (Table 1). The species were selected based on availability of their genome sequences, previous reports of their ability to produce trichothecenes, their close relationships to known trichothecene-producing species, and/or their representation of multispecies lineages of the genus *Trichoderma* (Kubicek et al., 2019).

**Table 1:** *Trichoderma* species and strains examined in the current study, and the occurrence of *tri5*, the *tri* cluster, and trichothecene production in the species/strains.

Lineage^a^	Species	Strain no.	Source of strain^b^	*tri5* ^c^	*tri* cluster^c^	Trichothecene production^d^	Reference^e^
*Brevicompactum*	*T. arundinaceum*	IBT 40837	DTU	+	+	harzianum A, trichodermol	Cardoza et al., 2011
	*T. aurantioeffusum* ^f^	S565	LWJ	−	−	ND	Current study
	*T. brevicompactum*	IBT 40841	DTU	+	+	trichodermin	Tijerino et al., 2011
	*T. margaretense* ^f^	S368	LWJ	+	−	ND	Current study
	*T. protrudens* ^f^	CBS 121320	WFBI	+	+	harzianum A	Current study
	*T. rodmanii* ^f^	CBS 121553	WFBI	+	+	trichodermin trichodermol	Current study
	*T. turrialbense* ^f^	CBS 112445	WFBI	+	+	harzianum A, isotrichodermin, trichodermol	Current study
*Trichoderma*	*T. asperellum*	CBS 433.97	na	+	−	None	Vicente et al., 2020 Current study
	*T. atroviride*	IMI 206040	na	−	−	None	N/A
	*T. gamsii* ^f^	T065	ULE	+	−	ND	Current study
	*T. hamatum*	GD12	na	−	−	None	N/A
	*T. koningiopsis*	P0S7	na	−	−	None	N/A
*Pachybasium*	*T. polysporum* ^f^	CBS 111723	WFBI	+	−	None	Current study
*Rubi*	*T. rubi* ^f^	CBS 127380	WFBI	+	+	ND	Current study
	*T. taxi* ^f^	TUCIM 2377	LID	+	+	trichodermin	Current study
*Deliquescens*	*T. deliquescens* ^f^	CBS 130572	WFBI	−	−	ND	Current study
*Psychrophila*	*T. balearicum* ^f^	CBS 133222	WFBI	+	+	roridin E	Current study
	*T. calamagrostidis* ^f^	CBS 121133	WFBI	+	+	ND	Current study
	*T. crystalligenum* ^f^	S38	LWJ	+	+	trichodermol, roridin A, E	Current study
	*T. oligosporum*	CGMCC 3.17527	na	+	+	roridin A, D, E trichodermadienediol	Chen et al., 2018
	*T. psychrophilum* ^f^	S647	LWJ	+	+	4-acetyltrichothecolone	Current study
	*T. rhododendri* ^f^	CBS 119288	WFBI	+	−	ND	Current study
*Hypocreanum*	*T. decipiens* ^f^	CBS 132861	WFBI	+	−	None	Current study
*Not assigned*	*T. albolutescens* ^f^	CBS 119286	WFBI	+	+	trichodermin, 16-hydroxytrichodermin	Ryu et al., 2017
*Semiorbis*	*T. cf. fertile* ^f^	CBS 137003	WFBI	+	−	ND	Current study
*Harzianum/Virens*	*T. atrobrunneum*	ITEM 908	na	−	−	None	N/A
	*T. guizhouense*	NJAU 4742	na	+	−	None	Vicente et al., 2020 Current study
	*T. harzianum*	CBS 226.95	na	−	−	None	N/A
	*T. lentiforme*	CFAM-422	na	−	−	None	N/A
	*T. virens*	Gv29-8	na	−	−	None	N/A
*Stromaticum*	*T. stromaticum* ^f^	CBS 101729	WFBI	+	−	ND	Current study
*Longibrachiatum*	*T. citrinoviride*	TUCIM 6016	na	−	−	None	N/A
	*T. koningii*	JCM 1883	na	−	−	None	N/A
	*T. parareesei*	CBS125925	na	−	−	None	N/A
	*T. reesei*	QM6a	na	−	−	None	N/A

a Subgeneric lineages of Trichoderma using names of Clades, Core Group and Sections described by Kubicek et al. (2019). The lineage Rubi was reported for the first time in this study.

b Sources of strains are indicated only for strains that were analyzed in laboratory experiments. DTU indicates Danish Technical University; LID indicates laboratory of Irina Druzhinina, TU Wien, Vienna, Austria; LWJ indicates the laboratory of Walter M. Jaklitsch at the University of Vienna, Vienna, Austria; ULE indicates the laboratory of Pedro A. Casquero and Santiago Gutiérrez at the University of León, Spain; WFBI indicates the Westerdijk Fungal Biodiversity Institute, Utrecht, The Netherlands; and na indicates that we examined only the genome sequence of the strain but did not acquire the strain for use in laboratory experiments.

c The symbol + indicates genes that were detected in a genome sequence by BLASTn analysis, and the symbol − indicates genes that were not detected in BLASTn analysis.

d ND indicates that no trichothecenes were detected using one or more of the analytical protocols described in the Methods section. None indicates that we inferred that the strain did not produce trichothecene based on the absence of *tri5* and/or other known *tri* genes in the genome sequence. However, trichothecene production was not assessed using analytical chemical methods.

e Publications in which trichothecene production was determined by analytical chemical methods. N/A indicates not applicable because trichothecene production has not been assessed be chemical analysis as far as we are aware.

f Species whose genome sequences were generated during the course of the current study. All other genome sequences were downloaded from the GenBank database (Supplementary Table S1a), except the sequences for *T. arundinaceum*, *T. brevicompactum* and *T. oligosporum*, which were generated as part of previous studies (Chen et al., 2018; Proctor et al., 2018).

All *Trichoderma* strains that were examined in laboratory experiments were sporulated on CMD medium (Yellow cornmeal 1g/L, potato dextrose broth 5g/L, Agar 1.5%), by incubation at 28°C for 10days, except for the strains of *T. balearicum*, *T. calamagrostidis*, *T. crystalligenum*, *T. psychrophilum*, *T. rhododendri* and *T. rubi*, which were incubated at room temperature (15–21°C) for longer periods (3–6weeks). *Rhizoctonia solani* strain ULE-R43, a fungal phytopathogen, was obtained from the University of León culture collection, and was used for antifungal assays on cellophane membranes. This strain was grown on potato dextrose agar medium (PDA) an incubated for 7days at 28°C in the dark.

### Nucleic Acid Extraction and Purification

#### Growth of Fungal Strains for Genomic DNA and RNA Purification

- For genomic DNA extraction: six plugs from each strain, collected from the CMD cultures described above, were inoculated in 100ml YPD medium (500ml flasks), and incubated for 24h at 28°C and 200rpm, except in the case of *T. balearicum*, *T. calamagrostidis*, *T. crystalligenum*, *T. psychrophilum*, *T. rhododendri*, and *T. rubi*, grown at 17°C, without shaking for 5–15days depending on the strain.
- For RNA extraction strains were grown following a similar procedure to that described above: six CMD plugs from each strain were inoculated in 100ml YPD medium and incubated for 24h at 28°C and 200rpm. However, in the case of *T. balearicum*, due to its low growth rate and to the specific conditions required for its growth, the CMD plugs were incubated on YPD for 24h at 28°C without shaking and later grown for 8 additional days at 17°C without shaking.
- Wild-type *T. arundinaceum* and strains derived from it were grown as described by Lindo et al. (2018) for RNA isolation. Briefly, 5×10^7^ spores from each strain were inoculated in 50ml of CM broth (0.5% malt extract, 0.5% yeast extract, AND 0.5% glucose) in 250-mL flasks, and incubated for 24h at 28°C with shaking at 250rpm. Ten milliliters of this pre-inoculum were transferred to 250ml flasks containing 50ml potato dextrose broth (PDB) medium and grown at 28°C with shaking at 250rpm. After 48h of incubation, mycelia were recovered by filtration through sterile Miracloth filters (Calbiochem, San Diego, CA), washed with 0.9% NaCl, freeze-dried, and used for DNA and/or RNA extraction.

#### Nucleic Acids Purification, and cDNA Synthesis

DNAs and RNAs were extracted from 20mg of grounded mycelia using the DNeasy Plant Mini Kit (QIAGEN, Hilden, Germany) and TRIZOL reagent (Invitrogen, Carlsbad, CA), respectively, as described previously (Lindo et al., 2019). After the isolation protocol, RNA was treated with RNase-free DNase and purified further through a Zymo-Spin column (Zymo Research, Irvine, CA). cDNA synthesis was carried out using the iScript™ cDNA synthesis kit (Bio-Rad, Hercules, CA) following the manufacturer’s instructions.

### Genome Sequences

Genome sequences from *T. arundinaceum*, *T. asperellum*, *T. atrobrunneum*, *T. atroviride*, *T. brevicompactum*, *T. citrinoviride*, *T. guizhouense*, *T. hamatum*, *T. harzianum*, *T. koningii*, *T. koningiopsis*, *T. lentiforme*, *T. parareesei*, *T. reesei*, *T. virens*, *Aspergillus hancockii*, *Beauveria bassiana*, *Cordyceps confragosa*, *Fusarium fasciculatum* (= phylospecies FIESC 12), *Fusarium graminearum*, *Fusarium longipes*, *Microcyclospora tardicrescens*, *Myrothecium roridum*, *Spicellum ovalisporum*, *Stachybotrys chartarum*, *Stachybotrys chlorohalonata*, and *Trichothecium roseum* were downloaded from the National Center for Biotechnology Information (NCBI) database (Supplementary Table S1). The genome sequences from the other 19 *Trichoderma* spp. examined were generated as a part of the current study using three different strategies. Strategy 1 was used for *T. albolutescens*, *T. aurantioeffusum*, *T. crystalligenum*, *T. decipiens*, *T. deliquescens*, *T.* cf. *fertile*, *T. margaretense*, *T. polysporum*, *T. protrudens*, *T. psychrophilum*, *T. rodmanii*, *T. stromaticum*, *T. taxi*, and *T. turrialbense*. For these species, genomic sequences were generated at USDA with a MiSeq Illumina platform (Illumina, Inc.), and were assembled with CLC NGS Cell v. 9.5 (Qiagen, Redwood City, CA) as previously described (Proctor et al., 2018). Strategy 2 was used for *T. calamagrostidis*, *T. rhododendri*, and *T. rubi*. For these species, genome sequences were generated by the company Macrogen (Seoul, Korea; https://dna.macrogen.com) using an Illumina platform, and sequences assembly was carried out by the SPAdes (v3.15.0) assembler (Bankevich et al., 2012). Strategy 3 was used for *T. gamsii* T065 and *T. balearicum*. For these species, genome sequences were generated by the company MicrobesNG (University of Birmingham, United Kingdom; https://www.microbesng.com) using similar procedures to those included in the strategy 2. For DNA extraction, strains whose genome was sequenced using strategies 2 or 3 were grown as described above. In the case of genomes sequenced following strategy 1, strains were grown as previously described (Proctor et al., 2018).

Genomes were annotated using the gene prediction software AUGUSTUS (Hoff and Stanke, 2013). Putative secondary metabolite biosynthetic gene clusters in the genomes were identified using the software antiSMASH 6.0 (Blin et al., 2021).

### Phylogenetic Analysis

#### Species Phylogenetic Tree

Nucleotide sequences from 20 *Trichoderma* housekeeping (=HK) genes (Supplementary Table S2) retrieved from the genome of 35 *Trichoderma* species (Table 1; Supplementary Table S1a) were used to infer a *Trichoderma* species tree. This study was carried out using two different methods, the results of which were combined. In Method 1, nucleotide sequences of the 20 HK genes from the *Trichoderma* species used in this work were retrieved as indicated above. Before the analysis, the introns from all genes were manually removed in order to have a unique continuous open reading frame in all of them. The sequences of each gene from all the *Trichoderma* spp. analyzed were individually aligned by MUSCLE software as implemented in MEGAX (Kumar et al., 2018), and then the alignments were concatenated using Sequence Matrix software (Vaidya et al., 2011). The resulting concatenated alignment was then subjected to maximum likelihood (ML) analysis as implemented in the program IQ-Tree version 1.6.7 (Nguyen et al., 2014). A second concatenated-partitioned tree was constructed by selecting for each gene the best-fit evolutionary nucleotide model deduced from the previous IQ-Tree analysis. Finally, both concatenated (non-partitioned and partitioned) alignments were subjected to ML analysis as implemented in IQ-Tree. Branch support was determined by bootstrap analysis using 1,000 pseudoreplicates. In Method 2, nucleotide sequences of each HK gene were aligned and then subjected to a ML analysis as indicated above. A consensus tree was then generated from the 20 individual HK trees as previously described (Stamatakis, 2014). Branch support in the consensus tree was determined by internode certainly (IC) as implemented in the program RAxML (Salichos et al., 2014).

#### *tri*5 Evolutionary Analysis

The presence or absence of *tri5* in the genome sequences of 35 *Trichoderma* species was determined by BLASTn analysis (Altschul et al., 1990) using the DNA sequence of *T. arundinaceum tri5* as a query. BLAST hits that were>80% identical to the query sequence were then aligned to previously described *tri5* DNA sequences from species of *T. arundinaceum*, *T. brevicompactum*, also from 11 non-*Trichoderma* species in the same order as *Trichoderma* (i.e., order Hypocreales), and from two outgroup species, *Microcyclospora tardicrescens* (order Capnodiales, class Dothideomycetes) and *Aspergillus hancockii* (order Eurotiales, class Eurotiomycetes), and a phylogenetic tree was then inferred from the alignment (Proctor et al., 2020). *tri5* sequences were aligned by the CLUSTAL software as implemented in MEGA7 (Kumar et al., 2016) and intron sequences were manually removed. The resulting ORFs were translated to amino acid sequences, aligned, and then converted back to the original nucleotide sequences before further analysis. Aligned *tri5* sequences were subjected to maximum likelihood (ML) analysis, and the best substitution model was determined with MEGA7 under the Akaike Information Criterion (AIC). The best model was GTR (General Time Reversible) with a discrete gamma distribution and an allowance for the presence of invariant sites (Nei and Kumar, 2000). Bootstrap analysis with 1,000 pseudoreplicates was used to determine the significance of nodes.

For assessments of horizontal gene transfer and selection, a tree was also inferred from the concatenated alignment of a subset of six housekeeping genes (*cpr1*, *dpa1*, *fas1*, *fas2*, *lcb2*, and *rpb1*; Supplementary Table S2) using the same procedure described above to infer the species tree. However, to avoid unwanted sources of variation, we used the same nucleotide substitution model that was used to infer the *tri5* tree to infer the six-housekeeping gene (6HK) tree. Branch support in the 6HK tree was assessed by both bootstrap and internode certainly analyses as described above.

#### Estimation of Divergence of *tri5*

To assess divergence and positive selection of *tri5* we compared estimates of the number of changes in codon sequences that change amino acid sequence (i.e., the number of nonsynonymous substitutions per nonsynonymous site, or dN) and changes in codon sequences that do not change amino acid sequence (i.e., synonymous substitutions per synonymous site, or dS). The estimates were obtained using the modified Nei-Gojobori model (Zhang et al., 1998) as implemented in MEGA7 (Kumar et al., 2016). We then estimated selection of *tri5* by determining the dN/dS ratio (**ω**) for pairwise combinations of species of (i) *Trichoderma* versus *Trichoderma* (TT), (ii) non-*Trichoderma* versus non-*Trichoderma* (NN), and (iii) *Trichoderma* versus non-*Trichoderma* (TN). **ω** values and statistics were obtained using the R statistics software package.

Positive selection was also assessed using CodeML as implemented in the program package PAML4 (Yang, 2007). Two models were compared with CodeML: (i) a null hypothesis (**H**_**0**_) in which **ω** was homogeneous in all branches in the *tri5* tree, and (ii) an alternative hypothesis (**H**_**1**_), in which **ω** differed in each branch in the *Trichoderma* clade of the *tri5* tree. The possible differences between these two models were statistically analyzed with the Kishino–Hasegawa test (Kishino and Hasegawa, 1989). The program BaseML, also included in PAML, was used to compare the different tree topologies versus a multiple alignment. Finally, the Shimodaira–Hasegawa test was used to determine if the topologies of the *tri5* and housekeeping gene trees were statistically different (pSH<0.05; Shimodaira and Hasegawa, 1999).

### Trichothecene Detection and Quantification

Trichothecene analogs and pathway intermediates were analyzed using three analytical systems: HPLC-UV, GC-MS, and LC-MS. The HPLC-UV system was used to detect and quantify harzianum A (HA) and consisted of a high performance liquid chromatography fitted with Waters YMC analytical column (150 by 4.6mm) and coupled to ultraviolet light absorption detector as previously described (Cardoza et al., 2011). The GC-MS system was used to detect and, in some cases, quantify all other simple trichothecenes and consisted of Hewlett Packard 6890 gas chromatograph fitted with a HP-5MS column (30m length, 0.25mm film thickness) coupled to an Agilent 5,793 mass detector as described previously (Lindo et al., 2018). The LC-MS system was used to detect macrocyclic trichothecenes and consisted of a ThermoDionex Ultimate 3000 liquid chromatograph fitted with a Phenomenex Kinetex F5 column (150mm length, 2.1mm diameter, 1.7μm particle size) coupled to the electrospray interface of a Thermo QExactive mass spectrometer operated in positive mode. The procedures for chromatographic separation and identification of trichothecene analogs were adapted from those previously described (Proctor et al., 2018). Together, the GC-MS, LC-MS, and HPLC systems used in this study had the capacity to detect over 150 trichothecene analogs (Cole et al., 1981; Savard and Blackwell, 1994; Lee et al., 2005; Nielsen et al., 2005; Degenkolb et al., 2008; Cardoza et al., 2011; Sun et al., 2016; Ryu et al., 2017; Chen et al., 2018).

For HPLC-UV analysis, strains were grown using the conditions described for RNA isolation described above. Filtrates from the resulting cultures were extracted with ethyl acetate. For the GC-MS and LC-MS analyses, strains were grown in liquid YEPD medium (0.1% yeast extract, 0.1% peptone, and 2% glucose) for 7days after which the resulting cultures (growth and culture medium) were extracted with ethyl acetate. All other experimental parameters (e.g., ratio of solvent volume to culture volume, injection volume, and chromatographic mobile phases) have been previously described (Cardoza et al., 2011; Proctor et al., 2018).

### Plasmid Construction

#### Construction of pΔtri5

1,180-bp and 1,061-bp fragments, corresponding to the 5'- and 3'-flanking regions to the *T. arundinaceum tri5* gene were amplified by PCR using the Q5 high-fidelity DNA polymerase (New England Biolabs, County Road, MA) and oligonucleotides tri5_5rF_BamHI/tri5_5rR_SmaI and tri5_3rF_SmaI/tri5_3rR_SalI, respectively (Supplementary Table S3). The amplicon corresponding to the 3'-flanking region was subcloned in pBluescript II KS+ (Stratagene, La Jolla, CA) previously digested with EcoRV, and dephosphorylated with alkaline phosphatase (Fermentas, Vilnius, Lithuania). The resulting plasmid, pBT5_3R (4,022bp) was digested with BamHI/SmaI and ligated to the tri5-5'-flanking amplicon, previously digested with the same endonuclease enzymes, to originate the plasmid pBT5_3R-5R (5,169bp). The resulting plasmid was linearized with SmaI, dephosphorylated, and then ligated to the *hygR* (hygromycin resistance) cassette (2,708bp), consisting of the coding region of the hygromycin phosphotransferase gene (*hph*) from *E. coli*, fused to the *gpdA* promoter and *trpC* terminator sequences of *A. nidulans*. HygR cassette was released from plasmid pAN71 (Punt et al., 1987) by HindIII digestion, treatment with Klenow fragment of DNA polymerase I (Fermentas), and a final digestion with Ecl136II. The resulting plasmid pΔtri5 (7,877bp) was used to delete *tri5* coding region of *T. arundinaceum* by a double cross-over strategy (Casqueiro et al., 1999).

#### Construction of Plasmids to Express the *tri5* Gene From Different *Trichoderma* Species Into the TARUN tri5 Gene Deleted Mutant (Δtri5.3)

The *tri5* ORFs were amplified by PCR using the Q5 high-fidelity DNA polymerase (New England Biolabs) from genomic DNAs of *T. arundinaceum* (1,226bp), *T. balearicum* (1,243bp), *T. decipiens* (1,176bp), *T.* cf. *fertile* (1,142bp), *T. gamsii* T065 (1,227bp), *T. polysporum* (1,223bp), and *T. stromaticum* (1,157bp), and 5'-phosphorylated oligonucleotides TARUN_T5_5/TARUN_T5_3, TBALE_T5_5/TBALE_T5_3, TDECI_T5_5/TDECI_T5_3, TFERT_T5_5/TFERT_T5_3, TGAMS_T5_5/TGAMS_T5_3, TPOLY_T5_5/TPOLY_T5_3, and TSTRO_T5_5/TSTRO_T5_3, respectively (Supplementary Table S3). The amplicons were subcloned in plasmid pTAcbh (Cardoza et al., 2015) previously digested with NcoI, filled with Klenow, and dephosphorylated. The resulting plasmids were linearized with EcoRI, except in the case of pTAcbh containing *T. arundinaceum tri5* that was digested with HIndIII. Then, plasmids were filled with klenow, dephosphorylated, and ligated to the 1,591-bp *bleR* (bleomycin/phleomycin resistance) cassette that was isolated and purified from plasmid pJL43b1 (Gutiérrez et al., 1997), by digestion with HindIII, filled with klenow, and again digested with Ecl136II. The final plasmids [pTC_T5TARUN_blea (8,164bp), pTC_T5TBALE_blea (8,181bp), pTC_T5TDECI_blea (8,114bp), pTC_T5TFERT_blea (8,080bp), pTC_T5TGAMS_blea (8,165bp), pTC_T5TPOLY_blea (8,161bp), pTC_T5TSTRO_bleb (8,095bp)] (Supplementary Figure S1b-h) were linearized with the endonuclease NdeI to transform protoplasts of the Δtri5-3 mutant. The *tri5* genes from the different *Trichoderma* strains used in this study, once subcloned as described above, were fully sequenced to confirm their integrity.

### *Trichoderma* Transformation and Selection of Transformants

Transformation of *T. arundinaceum* to obtain *tri5*-deletion mutants and transformants for heterologous expression of *tri5* homologs was carried out using a protoplast-mediated protocol as previously described (Proctor et al., 1999; Cardoza et al., 2006). Selection of *tri5*-deleted and *tri5*-complemented transformants was carried out on a regeneration medium containing hygromycin 150μg/ml or phleomycin 100μg/ml, respectively (Malmierca et al., 2012; Cardoza et al., 2019).

### Generation of a *T. arundinaceum* IBT 40837 *tri5*-Deleted Mutant (Δ*tri5*)

Deletion of *tri5* was achieved by transforming protoplasts of the *T. arundinaceum* strain IBT 40837 with plasmid pΔtri5 (7,877bp; Supplementary Figure S1a) that had been linearized with XhoI endonuclease. Colonies growing on the hygromycin B-amended regeneration medium were analyzed by the Terra PCR method (Takara Bio.) using two oligonucleotide primer pairs designed to detect homologous recombination between the deletion construct and the 5' (primer pair T5-5rr and TtrpC-d) and 3' (primer pair PgpdA-d and T5-3rr) flanking regions of *tri5* (Supplementary Table S3). Transformants that yielded both amplicons were also analyzed by PCR for the presence of a 674-bp fragment internal to *T. arundinaceum tri5*. Transformants that yielded the 674-bp amplicon were then subject to Sanger sequence analysis of the T5-5rr/TtrpC-d and PgpdA-d/T5-3rr amplicons to confirm that the amplicons originated from the expected double cross-over event. One transformant from which the expected sequence data for the 5' and 3' flanking regions was obtained was selected for genome sequence analysis, following procedure #1 described above, to further confirm deletion of the *tri5* coding region.

### Real Time qPCR

For qPCR analysis primers for *tri5* and actin-encoding genes were designed for all *Trichoderma* species analyzed in the present work (Supplementary Table S3). The primer pairs amplification efficiencies range between 91.3 and 110.6% (Supplementary Table S3). cDNAs were quantified using a Nanodrop ND-1000 (ThermoFisher), and qPCR reactions were carried out on a Step One system (Applied Biosystems) and the express SYBR green qPCR super-Mix Universal (Invitrogen) following manufacturer’s instructions. Finally, the qPCR Ct values were analyzing using the REST©2009 software (Pfaffl et al., 2002) to determine the expression ratio levels as well as the oligo pairs amplification efficiencies. Each measurement was done in triplicate.

### Volatile Collection and Characterization of Terpene Compounds Emitted From *Trichoderma* Cultures

Volatile compounds emitted from the transformants expressing the *tri5* from *T.* cf. *fertile* and *T. gamsii* T065 were collected using the previously described closed-loop stripping method (Laraba et al., 2020). Briefly, a 7-days old culture grown on potato dextrose agar (PDA) Petri dish (60mm×15mm) was sealed in a 3L glass desiccator. The air space within the desiccator was recirculated through a filter containing 25mg of Porapak™ Porous Polymer adsorbent, type Q, 80–100 mesh (Supelco, Bellefonte, PA, United States) for 24h at a rate of 3 volumes of total air space per hour. The compounds were then eluted from the adsorbent with 150μl of dichloromethane and 2μl of the effluent were analyzed on an Agilent 6890 chromatograph/Agilent 5973 mass spectrometer. The compounds were separated on a HP-5MS column held at 50°C for 3min after injection, and then the temperature was gradually heated to 250°C at 30°C/min where it was held for 1min. Individual peaks were identified based on comparison of ion fragmentation patterns. The terpene compounds were initially identified based on NIST 11 library spectral matches and then verified with standards.

### Antifungal Assays on Cellophane Membranes

These assays were performed as described previously (Cardoza et al., 2015), but incubating the *Trichoderma* plugs over the cellophane membranes for only 24h instead the 48h previously recommended. *Rhizoctonia solani* ULE-R43 was the pathogen used in these assays, and its growth was followed until the pathogen’s mycelium covers all the surface in the control plates, i.e., plates with the pathogen growing without previous growth of *Trichoderma*, which usually took 6–7days at 28°C in the dark. Three biological replicates were used for each *Trichoderma* strain.

## Results

### Phylogeny of the *Trichoderma* Species

**Figure 1:**
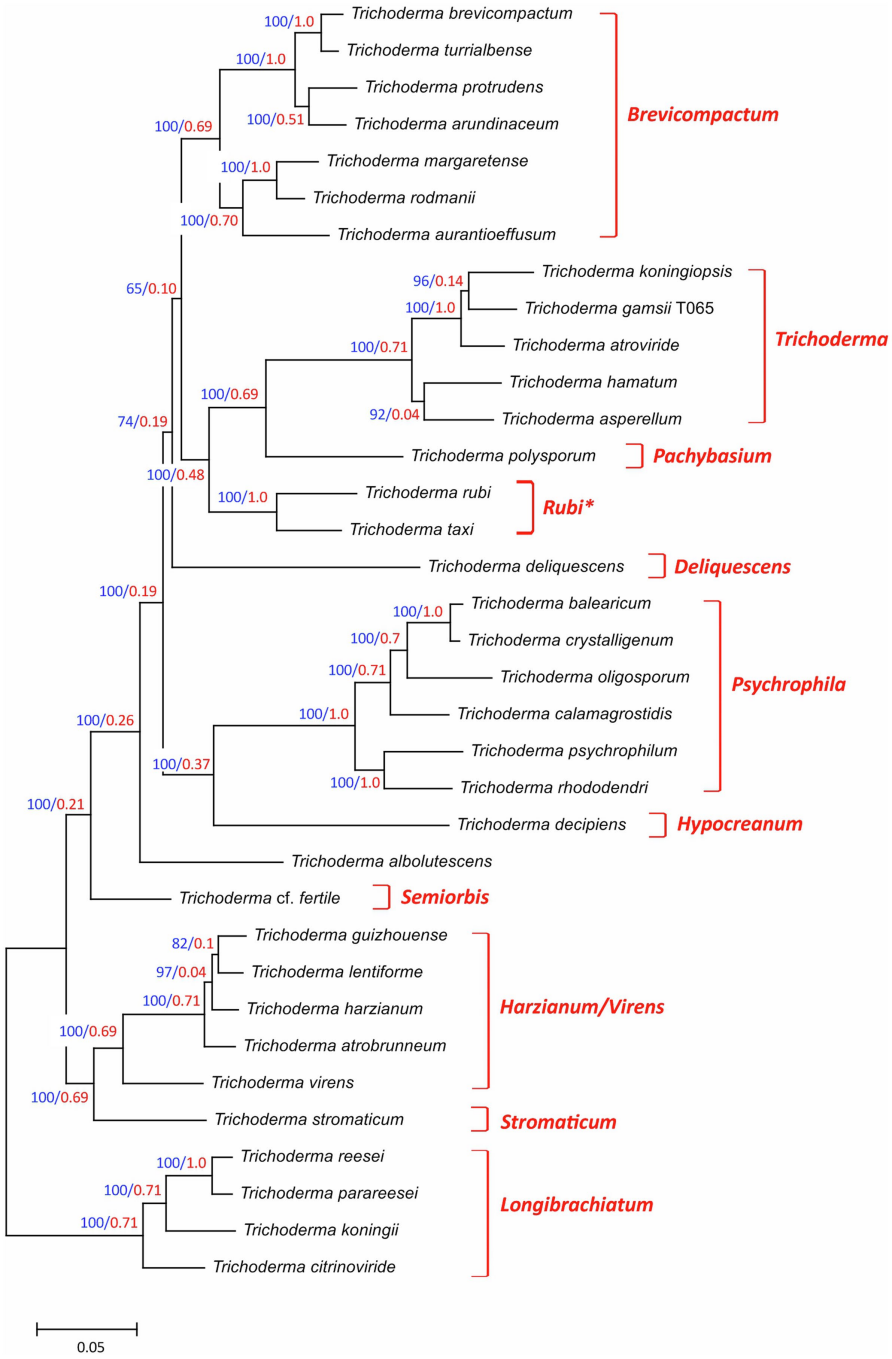
Species tree for the 35 *Trichoderma* species included in this study. The tree was inferred from full-length exon sequences of 20 housekeeping genes (= 52,200 total sites; Supplementary Table S2) as described in the Methods section. On each branch, numbers in blue type are bootstrap values based on 1,000 pseudoreplicates, and numbers in red type are internode-certainly values. The previously described subgeneric lineages (Kubicek et al., 2019) are indicated to the right of the tree. In the tree, the clade consisting of *T. rubi* and *T. taxi* has been assigned to a new lineage that we designated *Rubi*. *T. albolutescens* has not been assigned to a lineage. GenBank accession numbers of housekeeping gene sequences that were used in this analysis are included in Supplementary Table S4a (Note that In the table GenBank accession numbers are provided only for housekeeping gene sequences retrieved from genome sequences generated during the current study).

Together, the 35 *Trichoderma* species selected for this study represented 10 previously described multispecies lineages and three species (*T. albolutescens*, *T. rubi* and *T. taxi*) that were not previously assigned to lineages (Table 1). The previously described lineages *Brevicompactum*, *Longibrachiatum*, *Harzianum/Virens*, *Psychrophila*, *Trichoderma* were represented by 2–7 species each, while the previously described lineages *Deliquescens*, *Hypocreanum*, *Pachybasium*, *Semiorbis*, *Stromaticum* were represented by only one species each. To better understand the phylogenetic relationships of all 35 species, we inferred a species tree by maximum likelihood analysis of concatenated alignments of 20 housekeeping genes that were retrieved from genome sequences of the fungi (Figure 1). In the resulting tree, the five previously described lineages represented by multiple species were resolved into well supported and exclusive clades with bootstrap values of 100 and internode certainty values of 0.69–1.0 (Figure 1). For most other branches in the species tree, bootstrap values were 100 and internode certainty values ranged from 0.19 to 0.69. Only one branch had a bootstrap value less than 70: the branch that included the *Brevicompactum*, *Trichoderma*, and *Pachybasium* lineages as well as *T. rubi* and *T. taxi* (Figure 1). *T. rubi* and *T. taxi* were resolved as a well-supported clade, which we hereafter refer to as the *Rubi* lineage. The topology of the phylogenetic tree (Figure 1) was largely consistent with multiple species phylogenies that have been previously reported for other combinations of *Trichoderma* species (Jaklitsch and Voglmayr, 2015; Kubicek et al., 2019). Given this and the high levels of branch support, we used the species tree in Figure 1 to provide a phylogenetic context for *Trichoderma* species and lineages in subsequent analyses.

### Distribution of *tri5* Gene in Selected *Trichoderma* Species

**Figure 2:**
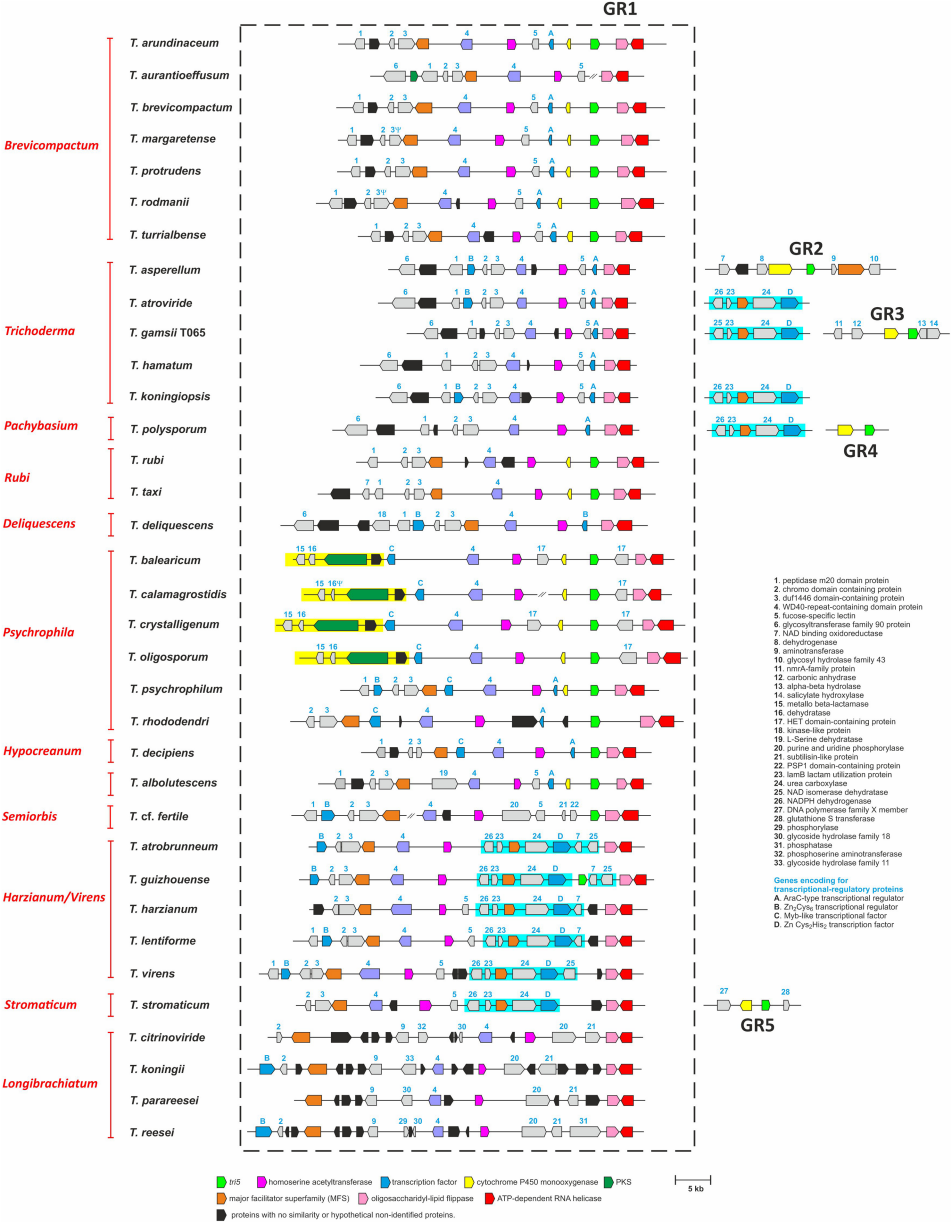
Analysis of the gene content in homologs of the *T. arundinaceum tri5* region (GR1) in 35 *Trichoderma* species. *tri5* was absent in GR1 homologs from 17 species and occurred at genomic locations other than GR1 in four species: GR2 in *T. asperellum*, GR3 in *T. gamsii* T065, GR4 in *T. polysporum*, and GR5 in *T. stromaticum*. Genes are represented by arrows that point in the direction of transcription. Numbers or letters above genes correspond to the numbers in the list of 37 proteins on the right side of the figure; the list indicates the predicted gene/protein functions based on sequence homology. Genes that are potentially involved in secondary metabolite biosynthesis and/or included in subsequent analyses are depicted as colored arrows. *tri5* is depicted as a bright green arrow; genes that do not exhibit homology to genes of known function are depicted as black arrows, and all other genes are depicted as gray arrows. Groups of arrows that are within a yellow or blue rectangle indicate multigene segments of DNA inserted into GR1. Names in red type on the left side of the figure are lineages of *Trichoderma* (Kubicek et al., 2019). The Greek letter ψ indicates that although a large portion of the gene is present, it has mutations that likely render it nonfunctional. GenBank accession numbers of genomic regions used in this analysis are included in Supplementary Table S4b (Note that in the table GenBank accession numbers are provided only for sequences of regions in genome sequences that were generated during the current study).

We used BLASTn analysis of genome sequences as an initial screen for the presence of *tri5* in the 35 *Trichoderma* species. This analysis indicated that a single copy of *tri5* was present in 22 species and absent in the other 13 species (Table 1; Figure 2). Of the 22 species with *tri5*, multiple similarities in the content and arrangement of flanking genes indicate that *tri5* is in the same genomic location, which we designated Genomic Region 1 (GR1), in 18 species and different genomic locations in each of four other species: GR2 in *T. asperellum*; GR3 in *T. gamsii* T065; GR4 in *T. polysporum*; and GR5 in *T. stromaticum*. Although the latter four species had *tri5*, results of BLASTn analysis indicated that they do not have any other known *tri* genes. Further, in all 22 species with *tri5*, the *tri5* flanking genes did not share significant sequence homology with known *tri* genes according to BLASTn and BLASTx analyses. This latter finding is consistent with the physical separation of *tri5* and the *tri* cluster that was previously reported for *T. arundinaceum* and *T. brevicompactum* (Cardoza et al., 2011; Proctor et al., 2018). The degree of conservation of genes in the *tri5*-flanking region was higher among species from the same lineage than those from different lineages (Figure 2). Based on sequence similarities to proteins with known functions, proteins encoded by some *tri5*-flanking genes included a cytochrome P450 monooxygenase, AraC-type transcriptional factor, major facilitator superfamily (MFS) transporter, homoserine acetyl transferase, oligosaccharidyl lipid flippase, and ATP-dependent RNA helicase, suggesting that some of the flanking genes could be involved in secondary metabolite biosynthesis while others are unlikely to be involved (Figure 2; Supplementary Table S5).

#### Trichothecene Production

**Figure 3:**
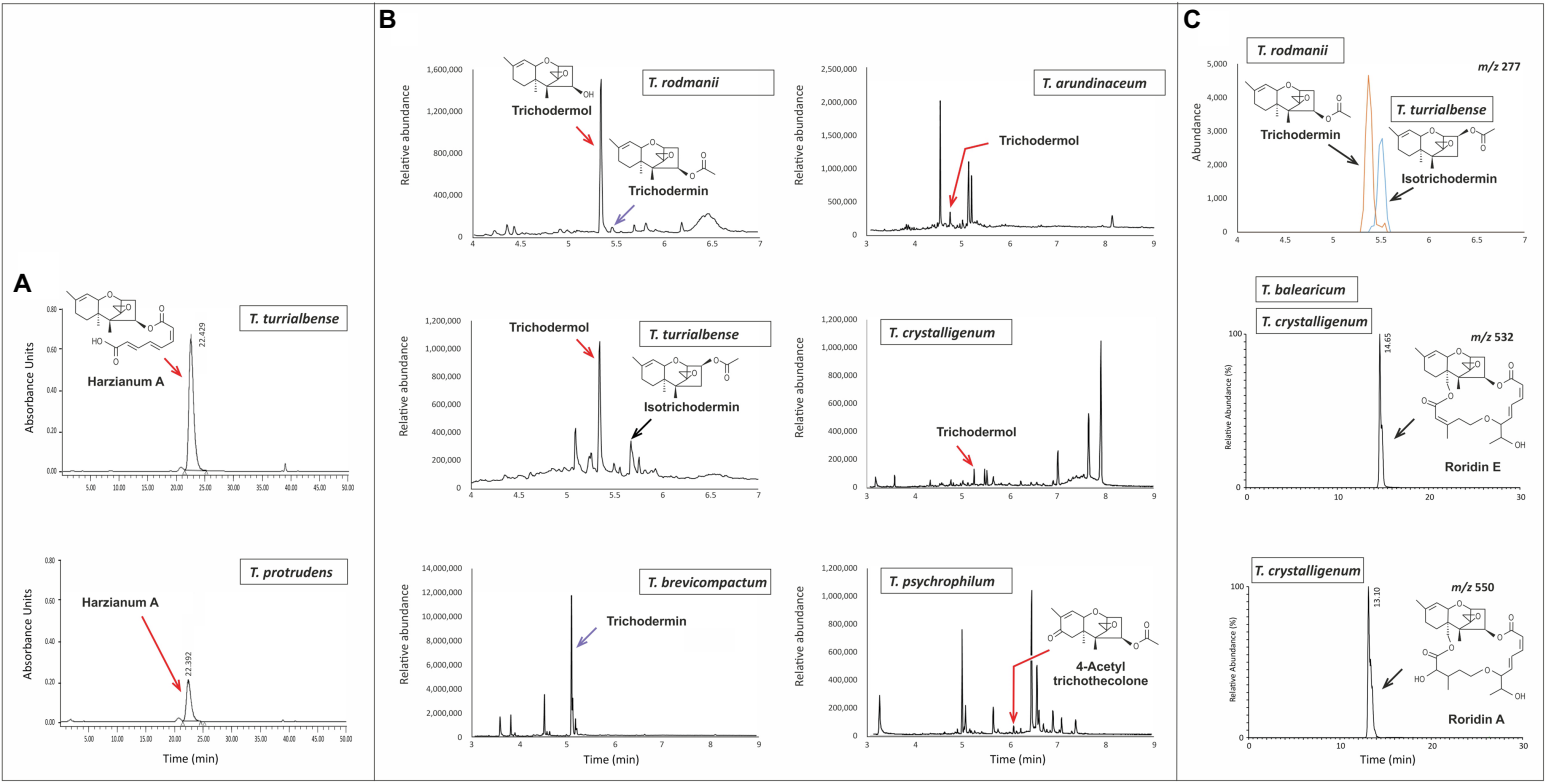
Analysis of trichothecene production in selected *Trichoderma* species. **(A)** Chromatograms from HPLC-UV analysis for determination of harzianum A production by *T. turrialbense* and *T. protrudens*. **(B)** Chromatograms from GC–MS analysis for production of multiple trichothecenes by *T. arundinaceum*, *T. brevicompactum*, *T. crystalligenum*, *T. psychrophilum*, *T. rodmanii*, and *T. turrialbense*. **(C)** Chromatograms from LC–MS analysis for detection of multiple trichothecenes: m/z 277 (upper panel) for detection of trichodermin and isotrichodermin production by *T. rodmanii* (5.4min peak) and *T. turrialbense* (5.5min peak); *m/z* 532 (middle panel) for detection of the [M+NH_4_]^+^ ion of roridin E produced by *T. balearicum* and *T. crystalligenum*; and *m/z* 550 (bottom panel) for detection of the [M+NH_4_]^+^ ion of roridin A produced by *T. crystalligeum*. Mass spectra for compounds shown in **(C)** are included in Supplementary Figure S2.

Our assessment of trichothecene production by *Trichoderma* species was limited by the availability of strains. Nevertheless, based on analyses in the current study and previously published reports, 11 out of the 35 species produced trichothecenes (Figure 3; Supplementary Figure S2; Table 1). Producing species were from the multispecies lineages *Brevicompactum* and *Psychrophila* as well as *T. albolutescens* and *T. taxi*. Of the seven species examined in the *Brevicompactum* lineage, five produced trichothecenes (Table 1). These five species produced one or more of the following simple trichothecene analogs: trichodermol, trichodermin, isotrichodermin, and harzianum A. Of the six species examined from the *Psychrophila* lineage, four produced trichothecenes. These species produced simple (e.g., trichodermin) or macrocyclic (e.g., roridins A, D, and/or E) trichothecenes or a mixture of both types of trichothecenes (Figure 3; Table 1). *T. taxi* produced trichodermin. Our analysis did not detect trichothecene production in *T. albolutescens* strain CBS 119286, which originated in Germany, but previous report indicated that another strain from Korea can produce trichodermin and 16-hydroxytrichodermin (Table 1; Ryu et al., 2017). We had genome sequences but not cultures for 11 *Trichoderma* species/strains and, therefore, could not analyze their trichothecene production profile. However, based on the absence of *tri5* and/or other *tri* genes in the genome sequences, we inferred that these strains could not produce trichothecenes (Table 1).

Production of trichothecenes by all species in the *Brevicompactum* lineage, except *T. margaretense* and *T. aurantioeffusum*, is consistent with the presence of *tri5* and other *tri* genes in the producing species and the absence of most or all of the genes in *T. margaretense* and *T. aurantioeffusum* (Table 1; Figure 3). In fact, when trichothecene production was initially described in *Trichoderma* species, it was proposed that production was limited to members of the *Brevicompactum* lineage (Cardoza et al., 2011). However, the results of the current study indicate that production, and presumably all necessary *tri* genes, occur in other lineages of the genus and occurs widely among members of in the *Psychrophila* lineage (Figure 2).

#### Variation in tri5 Genomic Region GR1

Analysis of genomic region GR1 revealed variation in the presence and absence of genes in homologs of the region in species from different *Trichoderma* lineages. There were two differences that stood out because they involved insertions of multigene segments of DNA into GR1.

The first multigene-segment insertion included 5–6 genes and occurred in members of the *Harzianum/Virens* and *Stromaticum* lineages (shaded in blue in Figure 2). In the segment, gene 24 was predicted to encode urea carboxylase (UC; Figure 2), which is thought to be involved in urea utilization (Navarathna et al., 2010). In the same segment, gene 26 encoded a NADPH dehydrogenase, gene 23 encoded a lactam utilization protein (LamB), gene MFS encoded a major facilitator superfamily, and gene D encoded a Zn Cys_2_His_2_ transcription factor. Whether these genes constitute a urea utilization gene cluster is not known. Homologs of these UC-related genes were also present and located adjacent to one another in *T. koningiopsis*, *T. gamsii* T065 and *T. atroviride* (*Trichoderma* lineage) and *T. polysporum* (*Pachybasium* lineage). However, in these latter four species, the UC-related genes were not located in GR1 (Figure 2).

The second multigene-segment insertion in GR1 occurred in four of six species in the *Psychrophila* lineage: *T. balearicum*, *T. calamagrostidis*, *T. crystalligenum*, and *T. oligosporum* (shaded in yellow in Figure 2). In Figure 2, only four genes in this segment are shown, but an in-depth analysis revealed that the segment consisted of 15 genes (Supplementary Figure S3). The segment also occurred in *T. psychrophylum* and *T. rhododendri*, which are also members of the *Psychrophila* lineage, but in these latter two species the segment was not located in GR1 (Supplementary Figure S3). Whether the 15 genes constitute a cluster is not known. However, a search for homologs of the segment using the antiSmash 6.0 software (Blin et al., 2021) revealed significant synteny with the agnestin biosynthetic gene cluster in *Paecilomyces divaricatus* (Szwalbe et al., 2019; Supplementary Figure S3).

### Phylogenetic Analysis of the *tri5* Gene

**Figure 4:**
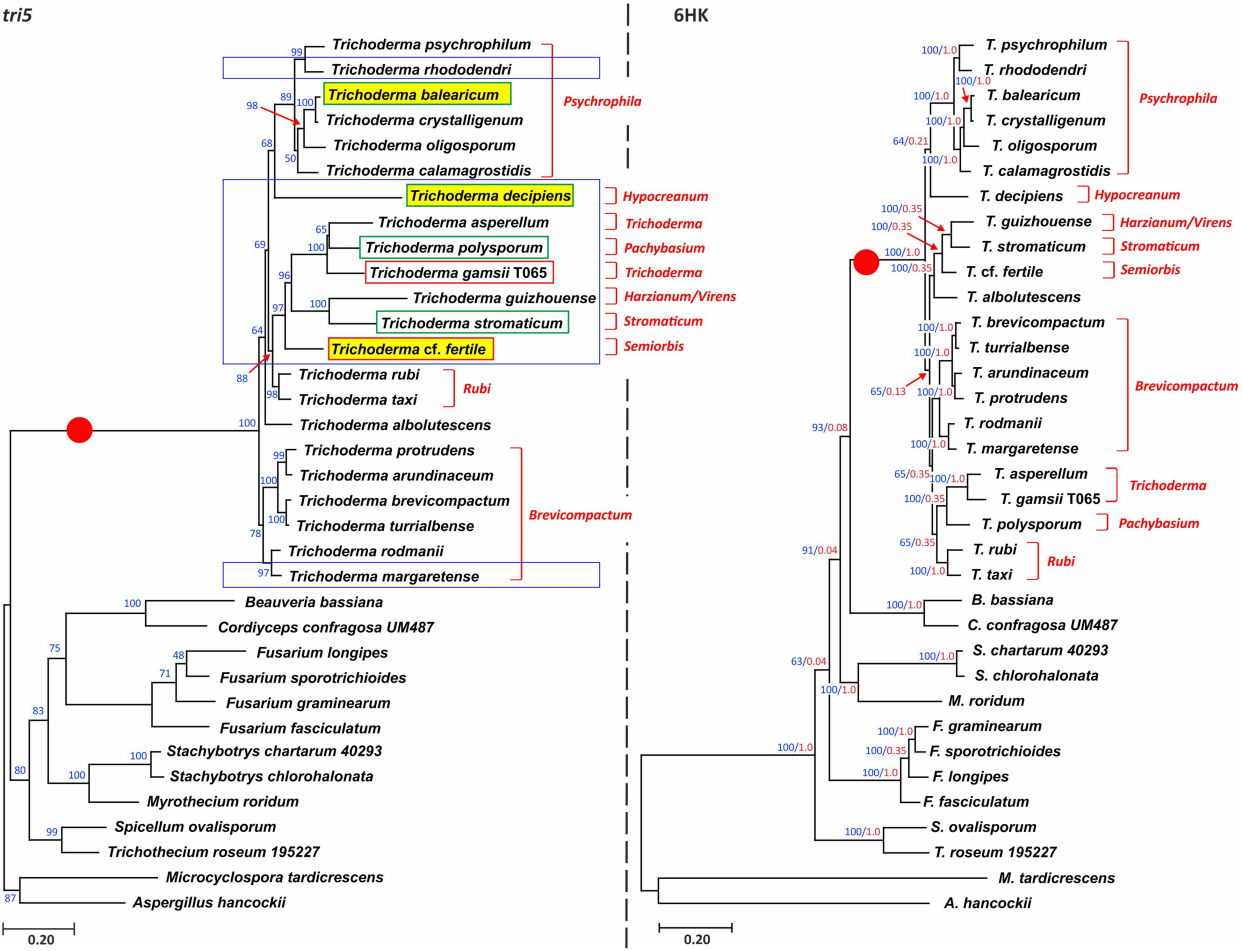
**(Left panel)** Phylogenetic tree inferred by maximum likelihood analysis of full-length exon sequences of *tri5* from *Trichoderma* species and other fungi. Numbers on each branch are bootstrap values based on 1,000 pseudoreplicates. Blue rectangles denote species that have *tri5* but no other *tri* genes. Red and green rectangles denote species used in the *Δtri5* mutant complementation analysis; a green rectangle indicates successful complementation, and a red rectangle indicates no complementation. Yellow highlighting denotes species that were both used in the complementation analysis and in which *tri5* is located at GR1. **(Right panel)** Phylogenetic tree inferred by maximum likelihood analysis of full-length exon sequences of six housekeeping genes (6HK; Supplementary Table S2) from the same species included in the *tri5* tree. Numbers on branches are bootstrap values (blue type) based on 1,000 pseudoreplicates and internode-certainly values (red type). In both panels, lineage names, as described previously (Kubicek et al., 2019), are indicated in red type to the right of each tree. The red circle in both trees denotes the different lengths of the *Trichoderma* branch in the two trees.

To assess the relationships of *tri5* homologs, we inferred a maximum likelihood tree from an alignment of full-length *tri5* exon sequences from 22 species of *Trichoderma* and 13 species from nine other fungal genera (Figure 4, left panel; Supplementary Table S1b). In the resulting tree, *Trichoderma tri5* sequences formed a well-supported and exclusive clade (bootstrap value=100; Figure 4). Within the *Trichoderma* clade, bootstrap support for nodes varied from <70 to 100. The topology within the *Trichoderma* clade was concordant with the species tree (Figure 4, right panel) in some respects but not in others. For example, the *Brevicompactum* and *Psychrophila* lineages formed monophyletic clades in both trees, although bootstrap values for these clades were 78 and 89, respectively, in the *tri5* tree but 100 in the species tree (Figures 1, 4). In contrast, although members of the *Trichoderma* lineage formed a well-supported and exclusive clade in the species tree, they were not monophyletic in the *tri5* tree. The relationships of *tri5* homologs from non-*Trichoderma* species were similar to those described in previous studies (Proctor et al., 2018, 2020). Another notable feature of the *tri5* tree was the long branch separating the *Trichoderma* clade from other non-*Trichoderma* clades (indicated with a red dot in Figure 4-left panel). This branch was longer than all other branches in the *tri5* tree and longer than the equivalent branch in the species tree (Figure 4, right panel; Supplementary Figure S4).

#### Analysis of tri5 Horizontal Transfer

Horizontal transfer of *tri5* to *Trichoderma* is one possible explanation for the branch conflicts in the species tree and the *tri5* tree as well as for the long branch leading to the *Trichoderma* clade in the *tri5* tree. In BLASTn and BLASTp analyses using *Trichoderma tri5* sequences as queries against the NCBI database, the highest-scoring hits were always *tri5* homologs from other *Trichoderma* species. In contrast, *tri5* homologs from other fungi had substantially lower BLAST scores and percent identity values. Thus, the results of BLAST analyses did not provide evidence for a potential donor in the putative horizontal transfer of *tri5* to *Trichoderma*.

To further investigate the possibility of horizontal transfer of *tri5* to *Trichoderma*, we used the Shimodaira-Hasegawa test to assess whether the *tri5* tree topology was significantly different than topologies of trees inferred from housekeeping genes. Our rationale was that if *tri5* had been horizontally transferred to *Trichoderma* from one of the other fungi included in this analysis, the Shimodaira-Hasegawa tests should indicate that the *tri5* tree topology was consistently different than the topology of all housekeeping gene trees. Therefore, in the analysis we inferred maximum likelihood trees from an alignment of *tri5*, individual alignments of six housekeeping genes, and a concatenated alignment of these six housekeeping genes. We then used the Shimodaira-Hasegawa test to determine whether the topology of each of the resulting (original) trees was more likely than topologies of trees inferred from the other alignments. The results of the analysis indicated that for each housekeeping gene alignment the original tree was more likely than the *tri5* tree topology (Table 2). The topology of the *tri5* alignment is rejected by all the HK alignments (pSH<0.05). However, the *tri5* alignment rejects (pSH<0.05) only the *fas1* and *lcb2* topologies, but it is compatible (pSH>0.05) with topologies of 6HK, *cpr1*, *dpa1*, *fas2*, and *rpb1* trees (Table 2). Thus, the results of this analysis indicate that the topology of the *tri5* tree was not significantly different from the topologies of all the housekeeping gene trees, a finding that does not support the proposed horizontal transfer of *tri5.*

**Table 2:** Probability values from a Shimodaira-Hasegawa (pSH) test that assessed whether the original maximum likelihood trees inferred from each sequence alignment was a better fit than trees inferred from other alignments.

	Alignment							
Topology	6HK	*cpr1*	*dpa1*	*fas1*	*fas2*	*lcb2*	*rpb1*	*tri5*
6HK	-	0.937	0.866	0.798	0.562	0.798	best	0.525
*cpr1*	0.398	-	best	0.864	0.548	0.752	0.300	0.416
*dpa1*	0.006**	0.214	-	0.100	0.036	0.506	0.251	0.107
*fas1*	0.000***	0.012*	0.001**	-	0.000***	0.000***	0.000***	0.000***
*fas2*	0.875	0.934	0.722	best	-	0.676	0.808	0.289
*lcb2*	0.000***	0.711	0.035*	0.027*	0.002	-	0.011*	0.034*
*rpb1*	0.001**	0.518	0.017*	0.281	0.001**	0.476	-	0.486
*tri5*	0.000***	0.000***	0.000***	0.000***	0.000***	0.000***	0.000***	-

A value of pSH<0.05 indicates that a tree inferred from another alignment was a significantly worse fit than the original tree.

One asterisk (*) indicates *p* values of 0.01–0.05, two asterisks (**) indicates p values of 0.0010–0.0099; and three asterisks indicate *p* values<0.001. Note that for all housekeeping gene alignments, the *tri5* tree topology can be discarded (pSH<0.05) with the data of any of the HK genes, i.e., it was a worse fit than the original housekeeping gene trees. However, the best topologies, 6HK, *cpr1* and *fas2* tree topologies, can not be discarded (pSH>0.05) by any of the alignments.

#### Assessment of *tri5* Selection

**Figure 5:**
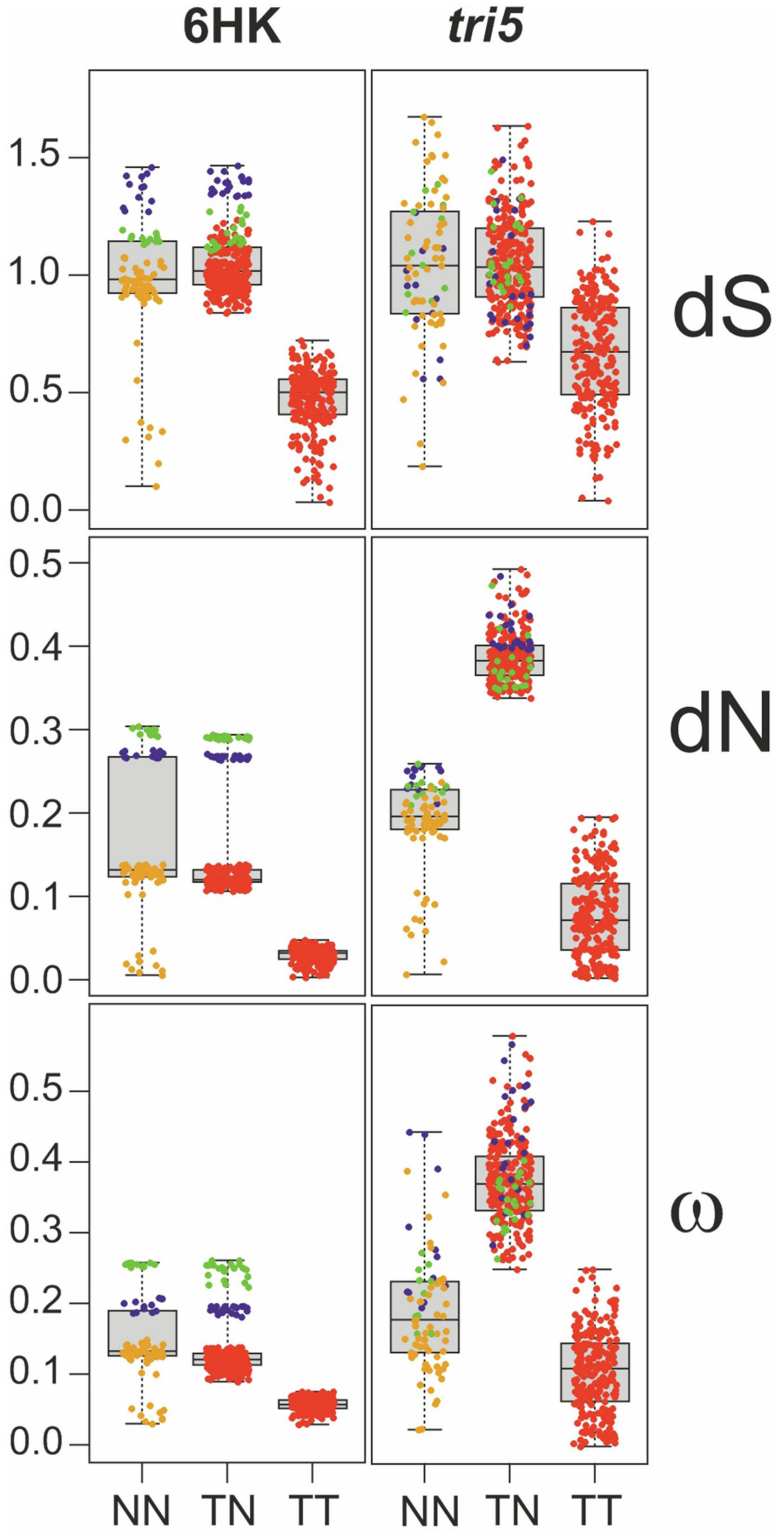
Boxplots and scatterplots of the distances between sequence pairs. The pairs analyzed were between non-*Trichoderma* (NN), *Trichoderma* and non-*Trichoderma* (TN), and *Trichoderma* (TT) genes. The distances of nonsynonymous changes per nonsynonymous site (dN); synonymous changes per synonymous site (dS), and the dN/dS ratio (ω) are included. The trees inferred from *tri5* and from concatenated alignments of six housekeeping genes (6HK) were considered in this analysis. Green and blue dots in the scatterplots indicate comparisons involving *Aspergillus hancockii* or *Microscyclospora tardicrescens* sequences, respectively. Red dots indicate comparisons involving all the other sequences. In the NN plot, however, orange dots indicate comparisons of non-Trichoderma species except for those that involve *Aspergillus hancockii* and *Microcyclospora tardicrescens*. Evidence for positive selection of *tri5*: marked shift upward of dN values for *Trichoderma* vs. non-*Trichoderma* (TN) comparisons in the *tri5* plot; this shift does not occur with dS values or in the 6HK plot.

The greater length of the *Trichoderma* branch relative to other branches in the *tri5* tree (Figure 4) could also result from rapid divergence of ancestral *Trichoderma tri5* after it diverged from other lineages of the gene. To assess whether this apparent rapid divergence resulted from positive selection of *tri5*, we compared estimates of the number of nonsynonymous substitutions per nonsynonymous site (dN), number of synonymous substitutions per synonymous site (dS), and the dN/dS ratio (=ω) for *tri5* and six housekeeping genes in pairwise combinations of species. We consolidated the comparisons into three types: i) non-*Trichoderma* versus non-*Trichoderma* (NN), ii) *Trichoderma* versus non-*Trichoderma* (TN) and iii) *Trichoderma* versus *Trichoderma* (TT; Figure 5).

The result of this analysis revealed that dN values for *tri5* were higher in *Trichoderma* vs. non-*Trichoderma* comparisons than in non-*Trichoderma* vs. non-*Trichoderma* comparisons (Figure 5; Table 3). A similar difference was observed for ω values for *tri5* in *Trichoderma* vs. non-*Trichoderma* comparisons relative to non-*Trichoderma* vs. non-*Trichoderma* comparisons (Figure 5; Table 3). The dN and ω values in *Trichoderma* vs. non-*Trichoderma* comparisons were also higher for *tri5* than for the housekeeping genes (Figure 5; Supplementary Figure S5), suggesting a higher rate of nonsynonymous changes in *tri5* than in the housekeeping genes. In contrast, the dN and ω values for *tri5* in *Trichoderma* vs. *Trichoderma* comparisons were not significantly different than for housekeeping genes, suggesting the rate of nonsynonymous changes in *tri5* slowed in *Trichoderma* species since they began diverging from one another. In contrast to the differences in dN and ω values noted above, *tri5* and housekeeping genes dS values were similar in *Trichoderma* vs. non-*Trichoderma* and non-*Trichoderma* vs. non-*Trichoderma* comparisons (Figure 5; Table 3; Supplementary Figure S5).

**Table 3:** Divergence values (mean±sd) for pairwise combinations of sequences.

	*cpr1*	*dpa1*	*fas1*	*fas2*	*lcb2*	*rpb1*	6HK	*tri5* *^a^*
dN								
NN	0.188 ± 0.083	0.208 ± 0.124	0.109 ± 0.058	0.136 ± 0.069	0.185 ± 0.104	0.196 ± 0.125	0.160 ± 0.088	0.187 ± 0.056
TN	0.199 ± 0.042	0.178 ± 0.085	0.111 ± 0.035	0.121 ± 0.045	0.191 ± 0.062	0.146 ± 0.097	0.143 ± 0.058	**0.386 ± 0.030**
TT	0.052 ± 0.018	0.029 ± 0.009	0.028 ± 0.009	0.022 ± 0.007	0.067 ± 0.031	0.011 ± 0.004	0.028 ± 0.009	0.076 ± 0.049
dS								
NN	0.946 ± 0.274	1.216 ± 0.290	0.906 ± 0.300	0.927 ± 0.309	1.064 ± 0.325	1.015 ± 0.262	0.988 ± 0.279	1.048 ± 0.308
TN	0.917 ± 0.153	1.269 ± 0.134	0.983 ± 0.180	0.985 ± 0.17	1.076 ± 0.15	1.138 ± 0.1	1.053 ± 0.132	1.055 ± 0.198
TT	0.411 ± 0.120	0.567 ± 0.157	0.446 ± 0.128	0.458 ± 0.127	0.482 ± 0.145	0.48 ± 0.131	0.475 ± 0.131	0.670 ± 0.234
ω								
NN	0.189 ± 0.073	0.162 ± 0.083	0.111 ± 0.040	0.135 ± 0.050	0.159 ± 0.064	0.176 ± 0.095	0.149 ± 0.060	0.19 ± 0.085
TN	0.221 ± 0.054	0.139 ± 0.057	0.113 ± 0.023	0.122 ± 0.031	0.177 ± 0.045	0.125 ± 0.073	0.134 ± 0.039	**0.376 ± 0.060**
TT	0.125 ± 0.027	0.052 ± 0.009	0.061 ± 0.012	0.049 ± 0.009	0.134 ± 0.045	0.021 ± 0.006	0.058 ± 0.009	0.107 ± 0.057

Comparisons were performed for pairs of sequences from two non-*Trichoderma* (NN), two *Trichoderma* (TT), or a *Trichoderma* and a non-*Trichoderma* (TN) species. Values are estimates of synonymous changes per synonymous site (dS); nonsynonymous changes per nonsynonymous site (dN), and the ratio of the two values (i.e., dN/dS=ω). Alignments of six housekeeping genes were included in this analysis. The trees inferred from *tri5* and the concatenated alignments of six housekeeping genes are shown in Figure 4.

a Values in bold type indicate *tri5* comparisons of *Trichoderma* versus non-*Trichoderma* pairs (TN) that yielded relatively high dN and **ω** values.

To further assess divergence of *tri5*, we used the program CodeML to compare two hypotheses: a null hypothesis (H_0_) in which ω was the same for all branches in the *tri5* tree, and an alternative hypothesis (H_1_) in which ω for the *Trichoderma* branch (ω_T_) differed from other branches (ω_0_) in the *tri5* tree. The results of this analysis revealed that the *Trichoderma* branch had a significantly higher ω than other branches in the *tri5* tree (Table 4). This in turn suggests positive selection within the *Trichoderma* branch of the *tri5* tree. A caveat to this result was that in the housekeeping gene tree the *Trichoderma* branch had a significantly higher ω than other branches. Nevertheless, the ω_T_/ω_0_ ratio in the *tri5* tree was substantially higher than in the housekeeping gene trees (Table 4).

**Table 4:** Maximum likelihood comparison between the hypotheses H_0_, based on a constant dN/dS ratio in the entire phylogenetic tree; and H_1_, with a different dN/dS ratio for the *Trichoderma* branch in the *tri5* or housekeeping gene trees.

	*cpr1*	*dpa1*	*fas1*	*fas2*	*lcb2*	*rpb1*	6HK	*tri5* ^a^
**H**_**0**_								
likelihood	−22,835	−58,581	−61,326	−70,279	−27,176	−61,566	−303,612	−19,111
**ω**_**0**_	0.12094	0.06462	0.06620	0.06127	0.10370	0.04781	0.06940	0.13967
**H**_**1**_								
likelihood	−22,827	−58,576	−61,322	−70,278	−27,173	−61,566	−303,598	−19,092
**ω**_**T**_	0.27162	0.10228	0.08885	0.07281	0.17410	0.05515	0.09243	0.59972
**ω**_**0**_	0.11705	0.06335	0.06509	0.06072	0.10158	0.04744	0.06837	0.13550
**ω**_**T**_ **/ω**_**0**_	2.321	1.615	1.365	1.199	1.714	1.163	1.352	**4.426**
**pKH**	0.024^*^	0.059	0.092	0.226	0.106	0.319	0.006^**^	**0.003** ^**^

The statistical differences between the likelihood values observed for each species pair was analyzed by the Kishino-Hasegawasa (pKH) test, using the program CodeML in the PAML software package.

a The value in bold text is the highest **ω** value observed in the *Trichoderma* branch (**ω**_**T**_) versus in all branches (**ω**_**0**_) in the *tri5* tree. ^*^Indicates *p* values of 0.011-0.049; and ^**^indicate *p* values < 0.01.

#### Phylogenetic Analysis of Trichoderma *tri5*-Flanking Genes

If the long *Trichoderma* branch in the *tri5* tree resulted from positive selection, it is possible that this selection affected neighboring genes through the phenomenon of genetic hitch-hiking (Barton, 2000). To determine whether the putative selection of *tri5* affected divergence of neighboring genes, we inferred trees from homologs of three *Trichoderma tri5*-flanking genes that encoded a putative homoserine acetyltransferase, a oligosaccharidyl lipid flippase, and a ATP-dependent RNA helicase (Figure 2). These three genes were selected because of their proximity to *tri5* in *Trichoderma* and the occurrence of closely related homologs in other fungi included in this study. In maximum likelihood trees inferred from the three genes, the *Trichoderma* branch was more similar in length to the *Trichoderma* branches in housekeeping gene trees than in the *tri5* tree. These results suggest that the putative positive selection on the *Trichoderma tri5* did not significantly affect divergence of the acetyltransferase, flippase, and helicase genes (Supplementary Figure S6).

### Analysis of Functionality of Selected *Trichoderma tri5* Homologs

The finding that some *Trichoderma* species have *tri5* but no other known *tri* genes (Table 1) raises the question about the functionality of the *tri5* in these species. Further, it is not known whether the different genomic locations of *tri5* can affect its function. To address these questions, we analyzed expression of the *tri5* homologs in their native locations and in a heterologous system.

#### Expression of tri5 in Selected *Trichoderma* Species

**Figure 6:**
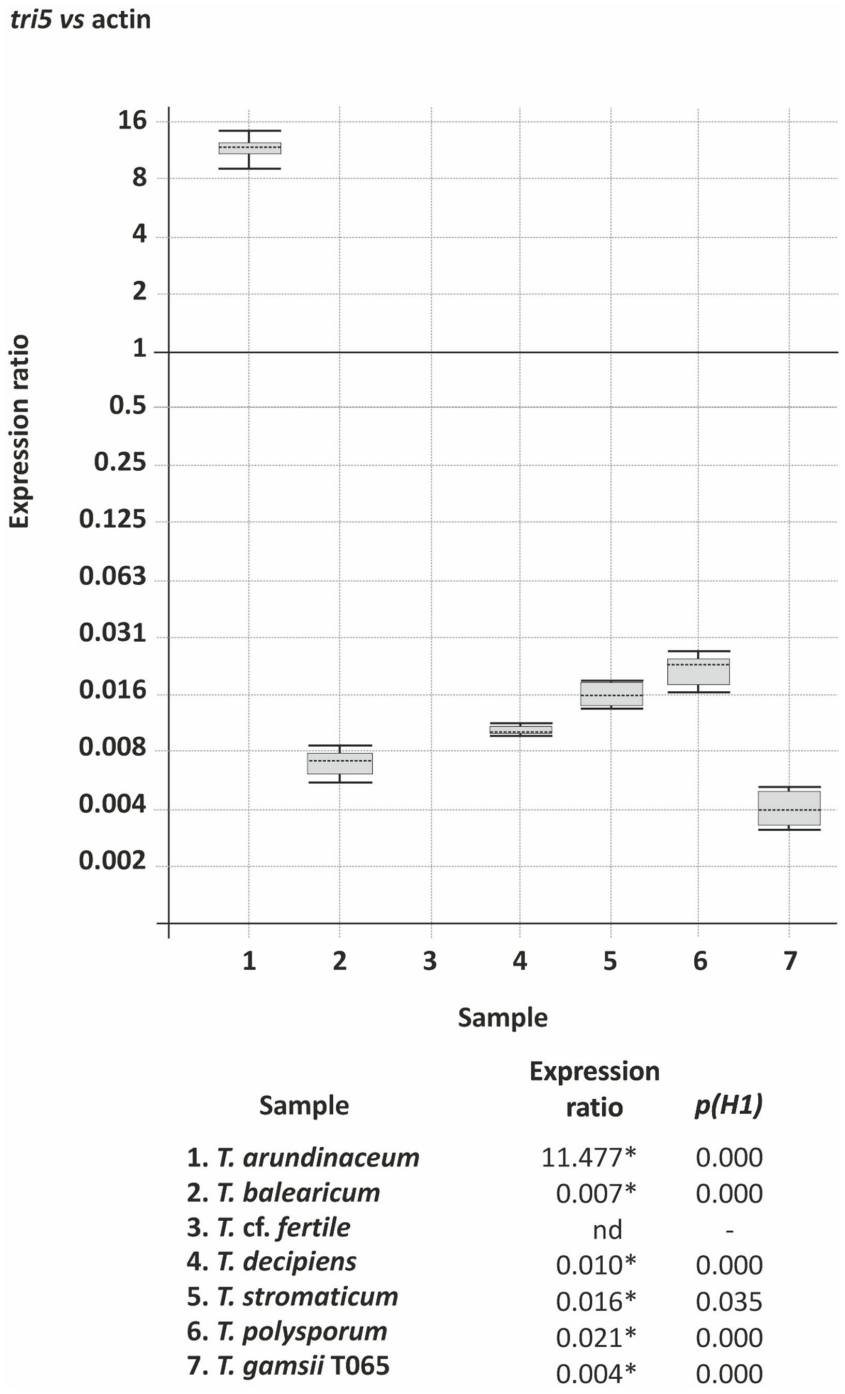
*tri5* expression in selected *Trichoderma* species. The data are derived from Real Time quantitative qPCR analysis and values are the level of expression of *tri5* versus the actin gene. Statistically significant expression ratio values [*p(H1)*<0.05] are indicated with an asterisk. nd indicates not detected.

We compared expression of *tri5* in the following selected *Trichoderma* species: i) *T. arundinaceum* and *T. balearicum*, which produce trichothecenes, have a *tri* cluster, and have *tri5* in genomic region GR1; ii) *T.* cf. *fertile* and *T. decipiens*, which do not produce trichothecenes, lack the *tri* cluster, and have *tri5* in genomic region GR1; and iii) *T. gamsii* T065, *T. polysporum*, and *T. stromaticum*, which do not produce trichothecenes, lack the *tri* cluster, and have *tri5* in genomic region GR3, GR4 and GR5, respectively. The results of the analysis indicated high levels of *tri5* expression in *T. arundinaceum*, no detectable expression in *T.* cf. *fertile*, and very low levels of expression in the other five species examined (Figure 6). Surprisingly, *tri5* expression in *T. balearicum* was very low even though it produced the trichothecene roridin E under the same conditions (Table 1; Figure 3).

#### Development of Heterologous tri5 Expression System

**Figure 7:**
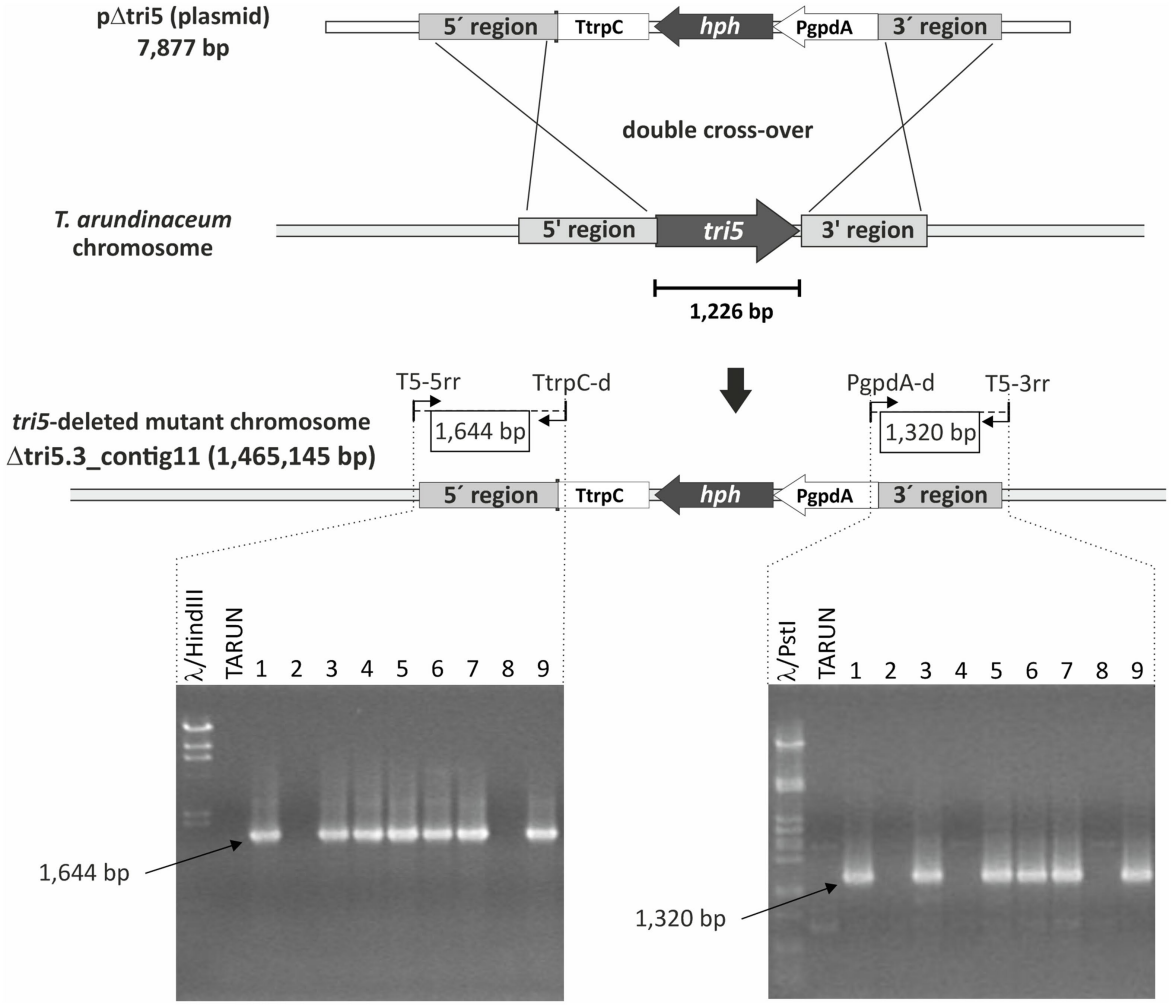
Scheme of the strategy used to delete *tri5* homolog in *T. arundinaceum* IBT 40837. Gel images below diagram of deletion strategy show PCR products amplified by the primers shown in the scheme.

We rationalized that a *tri5* deletion mutant (Δ*tri5*) of *T. arundinaceum* would serve as an effective heterologous expression system for *tri5* homologs from other *Trichoderma* species, because the mutant should not produce trichothecenes, but functional homologs of *tri5* should complement the mutant to restore trichothecene production. Using the deletion protocols described in the Methods we identified three Δ*tri5* mutants of *T. arundinaceum* (Figure 7). None of them produced detectable levels of harzianum A under conditions that induced production of high levels of the trichothecene in the wild-type progenitor strain (Supplementary Figure S7). Genome sequence analysis of one of the mutants, strain Δtri5.3, confirmed *tri5* deletion. Therefore, we selected strain Δtri5.3 as the host for heterologous expression analyses.

#### Heterologous Expression of *tri5* Homologs From Selected *Trichoderma* Species

For heterologous expression analysis, we selected *tri5* homologs from *T. balearicum*, *T.* cf. *fertile*, *T. gamsii* T065, *T. decipiens*, *T. polysporum*, *T. stromaticum*, and *T. arundinaceum* (positive control). A separate *tri5* overexpression plasmid was constructed for each of these *tri5* homologs, and in each plasmid the *tri5* coding region was fused to the *T. harzianum*-*tadir* gene promoter, which confers high levels of expression in *T. arundinaceum* (Proctor et al., 2018; Cardoza et al., 2019; Lindo et al., 2019; Supplementary Figures S1b–h). Each plasmid was transformed separately into Δ*tri5* mutant strain Δtri5.3, and 20 of the resulting transformants recovered for each construct were analyzed by PCR to verify integration of the *tri5*-expression cassette. Two transformants for each construct that yielded the expected amplicons were then selected for trichothecene analysis. Transformants carrying *tri5* homologs from *T. arundinaceum*, *T. balearicum*, *T. decipiens*, *T. stromaticum*, and *T. polysporum* produced harzianum A (HA), but the levels produced ranged from 0.3 to 30.0 percent of levels produced by wild-type *T. arundinaceum* (Table 5). Thus, in some species that lack other *tri* genes (e.g., *T. stromaticum*, *T. decipiens*, and *T. polysporum*) *tri5* has retained its function in trichodiene production (Table 5). In contrast, transformants carrying *tri5* homolog from *T.* cf. *fertile* or *T. gamsii* T065 did not produce detectable levels of HA or any of the other more than 150 trichothecene analogs detected by our analytical systems. We analyzed a total of seven transformants each with the *T.* cf. *fertile* and *T. gamsii* T065 homologs, and none produced detectable levels of trichothecenes (Table 5; Supplementary Figure S7). These findings indicate that in *T. cf. fertile* and *T. gamsii* T065, which lack other *tri* genes, the *tri5* gene has not retained its function in trichodiene biosynthesis.

**Table 5:** Harzianum A (HA) production by transformants of the *T. arundinaceum tri5* deletion mutant (strain Δtri5.3) expressing *tri5* homologs from other *Trichoderma* species.

Strain	Origin of *tri5* homolog	HA (μg/ml)	% production of positive control^a^
wild-type *T. arundinaceum*	*T. arundinaceum*	168.67 ± 1.87	100
Δtri5.3	None	nd	0
ΔT5_T5TARUN 24	*T. arundinaceum*	34.47 ± 2.12	20.43
ΔT5_T5TARUN 30	*T. arundinaceum*	51.49 ± 0.71	30.52
ΔT5_T5TBALE 2	*T. balearicum*	0.45 ± 0.03	0.27
ΔT5_T5TBALE 3	*T. balearicum*	32.15 ± 6.83	19.06
ΔT5_T5TDECI 2	*T. decipiens*	3.12 ± 0.28	1.85
ΔT5_T5TDECI 3	*T. decipiens*	2.53 ± 0.22	1.50
ΔT5_T5TFERT 2	*T.* cf. *fertile*	nd	0
ΔT5_T5TFERT 3	*T.* cf. *fertile*	nd	0
ΔT5_T5TGAMS 6	*T. gamsii* T065	nd	0
ΔT5_T5TGAMS 10	*T. gamsii* T065	nd	0
ΔT5_T5TPOLY 1	*T. polysporum*	12.62 ± 0.57	7.48
ΔT5_T5TPOLY 2	*T. polysporum*	11.39 ± 0.01	6.75
ΔT5_T5TSTRO 4	*T. stromaticum*	30.10 ± 2.3	17.84
ΔT5_T5TSTRO 5	*T. stromaticum*	28.08 ± 4.98	16.65

a Percent of HA level relative to level produced by wild-type *T. arundinaceum*.

**Figure 8:**
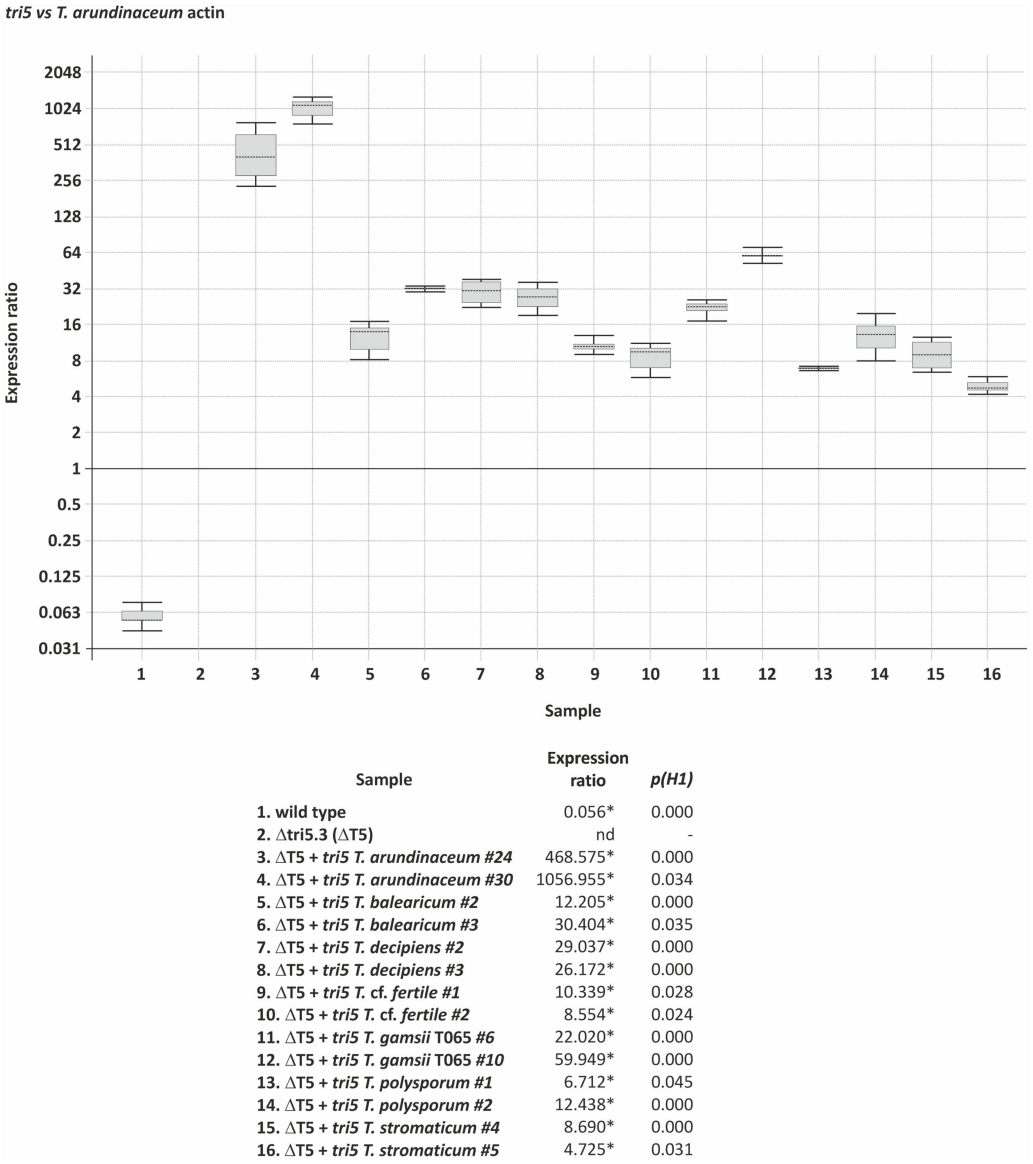
*tri5* expression in *T. arundinaceum* strains generated in the complementation analysis. Values are derived from Real Time quantitative PCR (qPCR) analysis and are the ratio of the level of expression of *tri5* versus the actin gene. Strains: wild type – wild-type *T. arundinaceum*, and Δtri5.3 (Δ5) – *T. arundinaceum Δtri5* deletion mutant. The 14 other strains are transformants of Δtri5.3 expressing *tri5* homologs from seven *Trichoderma* species. Statistically significant expression ratio values [*p(H1)*<0.05] are indicated with an asterisk. nd indicates not detected.

We confirmed expression of the *tri5* homologs in transformants of Δtri5.3 using qPCR analysis with RNAs extracted from mycelia obtained from the HA production analysis. The results of the analysis indicate that the *tri5* homologs from all seven donor species were expressed at high levels (Figure 8). Thus, transformants carrying the *T.* cf. *fertile* and *T. gamsii* T065 homologs that did not produce trichothecenes had high levels of *tri5* expression.

#### Production of Volatiles in Heterologous Expression Systems

To determine whether the *T.* cf. *fertile* or *T. gamsii* T065 *tri5* homologs confer production of a terpene(s) other than trichodiene, we analyzed Δtri5.3 transformants expressing these *tri5* homologs. Thus, if the *T.* cf. *fertile* and *T. gamsii* T065 TRI5 homologs catalyze synthesis of a terpene other than trichodiene, the terpene should be detected as a volatile. However, GC–MS analysis of the headspace of cultures did not reveal any qualitative differences in volatiles produced by (i) transformants expressing the *T.* cf. *fertile* or *T. gamsii* T065 *tri5* homolog, (ii) untransformed control strain Δtri5.3, and (iii) wild-type *T. arundinaceum*, except that the wild type produced trichodiene while the transformants and Δtri5.3 did not. These results do not provide evidence that the *T.* cf. *fertile* and *T. gamsii* T065 *tri5* homologs confer production of trichodiene or another volatile terpene.

#### Analysis of Trichodiene Production by *T. decipiens*, *T. polysporum*, and *T. stromaticum*

In the heterologous expression analysis, the *T. decipiens*, *T. polysporum and T. stromaticum tri5* homologs complemented the *tri5* mutation in *T. arundinaceum*, indicating that the homologs are functional. To further examine their function, we determined whether wild-type strains of *T. decipiens*, *T. polysporum*, and *T. stromaticum* produced trichodiene using the headspace assay described for the previous section. No trichodiene was detected in the head space from cultures of the three species. Thus, even though the *T. decipiens*, *T. polysporum*, and *T. stromaticum tri5* homologs are functional, activity of the corresponding enzymes could not be detected in the native strains. A likely explanation for this is the extremely low level of *tri5* expression in the three species under the growth conditions used in our experiment (Figure 6).

#### Analysis of the Antifungal Activity

Production of trichothecenes has been shown to contribute to the ability of *T. arundinaceum* and *T. brevicompactum* to inhibit the growth of some plant pathogenic fungi. Therefore, we examined the ability of other trichothecene-producing and nonproducing species/strains of *Trichoderma* to inhibit the growth of the plant pathogen *Rhizoctonia solani*. In all experiments, the *T. arundinaceum* Δ*tri5* mutant, strain Δtri5.3, caused markedly less growth inhibition than its wild-type progenitor strain, indicating that trichothecene production contributes substantially to the antifungal activity of *T. arundinaceum* against *R. solani* (Supplementary Figures S8, S9).

In one set of experiments, we assessed antifungal activity of wild-type strains of four species that have *tri5* but no other *tri* genes: *T. decipiens*, *T. gamsii* T065, *T. polysporum*, and *T. stromaticum*. All four species inhibit *R. solani* growth much less than wild-type *T. arundinaceum* (Supplementary Figure S8). Growth inhibition caused by *T. decipiens* was similar to that caused by Δtri5.3, while inhibition caused by *T. gamsii* T065, *T. polysporum*, and *T. stromaticum* was less than that caused by Δtri5.3.

In a second set of experiments, we examined the effect that expression of *tri5* homologs from trichothecene-producing and nonproducing species of *Trichoderma* had on the antifungal activity of strain Δtri5.3. The antifungal activity of Δtri5.3 transformants expressing *tri5* homologs was correlated with the levels of HA produced by them (Supplementary Figure S9). That is, transformants expressing the *T. arundinaceum* (positive control), *T. balearicum*, and *T. stromaticum tri5* homologs, which produced relatively high levels of trichothecenes, caused the highest levels of inhibition, while transformants expressing the *T. decipiens* and *T. polysporum tri5* homologs, which produce lower levels of HA, cause less growth inhibition (Supplementary Figure S9). Further, transformants expressing *tri5* homologs from *T.* cf. *fertile* and *T. gamsii* T065, which did not produce HA, caused levels of inhibition similar to those caused by strain Δtri5.3 (Supplementary Figure S9).

## Discussion

### Distribution and Location of *tri5* in *Trichoderma* Lineages

Various combinations of *tri* genes sufficient to confer trichothecene biosynthesis have been reported in 14 fungal genera of the Ascomycota (Proctor et al., 2020), and trichothecene production has been confirmed in species from 10 of these genera, including at least six species of *Trichoderma* (Cardoza et al., 2011; Tijerino et al., 2011; Dawidziuk et al., 2014; Sun et al., 2016; Chen et al., 2018). In the current study, examination of genome sequences of 35 *Trichoderma* species revealed that *tri5* and the *tri* cluster occur in diverse *Trichoderma* species but that *tri5* is more common than the *tri* cluster (Table 1). The results also indicated that physical separation of *tri5* and the *tri* cluster is a conserved character among *Trichoderma* species that have both loci. It is noteworthy that in 18 *Trichoderma* species, *tri5* was located at the same genomic location (GR1) as originally described in *T. arundinaceum* and *T. brevicompactum*, whereas the occurrence of *tri5* in other genomic location was much less common. Nevertheless, its occurrence at GR2–GR5 suggests that *tri5* has undergone two translocation events during divergence of *Trichoderma* species. However, our understanding of GR2–GR5 was limited by the small contigs on which these regions occurred. Although our analyses demonstrated conservation of the physical separation of *tri5* and the *tri* cluster in *Trichoderma*, the biological significance of the separation is not clear (Proctor et al., 2020).

The 35 *Trichoderma* genome sequences examined in the current study represent 12 phylogenetically distinct lineages of the genus *Trichoderma*, and the species tree inferred in the current study (Figure 1) was correlated with previously reported species trees (e.g., Kubicek et al., 2019). Analysis of the presence and absence of *tri5* in the context of the species tree revealed that the gene was present in all lineages examined except *Deliquescens* and *Longibrachiatum* (Table 1). Within the 10 lineages with *tri5*, its occurrence varied from present in all species examined (e.g., *Psychrophila*), present in most species (*Brevicompactum*), and present in a minority of species (*Harzianum/Virens*). This variation in occurrence suggests that *tri5* has been lost multiple times in *Trichoderma* and potentially multiple times within some lineages. However, the results do not rule out the possibility that horizontal transfer between *Trichoderma* species has contributed to variation in occurrence of *tri5* within and among lineages. Regardless, its widespread distribution among the lineages examined suggests that *tri5* was present in *Trichoderma* prior to divergence of the lineages.

*Fusarium* trichothecenes are an important agricultural and food/feed safety concern because of their toxicity and frequent occurrence in crops (Munkvold et al., 2021). A comparison of results from the current study on *Trichoderma* and previous studies on *Fusarium* indicate marked difference in the presence and absence of *tri* genes and trichothecene production in the two genera. When the genus *Trichoderma* is viewed as a whole, the distribution of *tri5*, the *tri* cluster, and trichothecene production are discontinuous. In *Fusarium* by contrast, the presence of the *tri* cluster, which includes *tri5*, and trichothecene production is continuous in two closely related, multispecies lineages (the *F. incarnatum*-*equiseti* and *F. sambucinum* species complexes), whereas the cluster and production are absent in 20 other lineages (Proctor et al., 2009; O’Donnell et al., 2013; Villani et al., 2019; Brown et al., 2020; Laraba et al., 2021). A potential caveat is that *Fusarium* may encompass more phylogenetic diversity than *Trichoderma* (Crous et al., 2021; Geiser et al., 2021). Nevertheless, the *F. incarnatum*-*equiseti* and *F. sambucinum* species complexes comprise at least 35 and 70 phylogenetically distinct species, respectively, in which the *tri* cluster appears to be continuously distributed (Xia et al., 2019; Laraba et al., 2021). Based on our current understanding, *tri5* and the *tri* cluster do not have a continuous distribution over similar numbers of phylogenetically distinct *Trichoderma* species. This difference in distribution suggests differences in selection to retain *tri* genes in the two genera. What the drivers of such differences in selection would be are not clear, but one tempting argument is that *Fusarium* species tend to be plant pathogens while *Trichoderma* species tend to be saprobes. This argument falls short, however, because some members of the *F. incarnatum*-*equiseti* and *F. sambucinum* complexes are weak plant pathogens at best, and some other *Fusarium* species complexes include species that are highly aggressive plant pathogens but lack *tri* genes (Leslie and Summerell, 2006; Munkvold et al., 2021).

### Evidence for a Positive Selection of *tri5* in *Trichoderma* spp.

The results of the current study also provide evidence for positive selection of the ancestral *Trichoderma tri5* during its divergence from *tri5* in other fungi (Figures 4, 5). There is evidence for positive selection of other secondary metabolite biosynthetic genes in fungi (Massonnet et al., 2018; Alouane et al., 2021). In these other fungi, the selection was attributed to adaptation to environmental changes. Such adaptation could have also driven positive selection of *tri5*, but it is not clear what the specific driver(s) would be. Three other observations could provide further insight into the positive selection and other aspects of the evolution of *Trichoderma tri5*. First, as noted previously, *tri5* and the *tri* cluster are at different genomic locations in *Trichoderma*. Further, there is no evidence for positive selection of *Trichoderma tri* cluster genes; the *Trichoderma* branch in trees inferred from each of six *tri* cluster genes is markedly shorter than in the *tri5* tree (Proctor et al., 2018). Their physical separation and different rates of divergence suggest that the *Trichoderma tri5* and *tri* cluster have had distinct evolutionary histories. Second, in the *tri5* tree, branch lengths within three single-genus clades, *Trichoderma*, *Fusarium*, and *Stachybotrys*, are generally similar, although there are some differences (Figure 4). This similarity suggests that the positive selection occurred on the ancestral *Trichoderma tri5* but was relaxed as *Trichoderma* species diverged from one another. Third, we surmise that the location of *tri5* at GR1 is ancestral to its location at GR2–GR5, because *tri5* occurs at GR1 in seven diverse *Trichoderma* lineages, while *tri5* occurs in GR2–GR5 in only three lineages, two of which are closely related (Figure 2). Further, despite evidence that positive selection on a gene can affect divergence of neighboring genes (Barton, 2000), we found no evidence for positive selection of three *tri5*-flanking genes at GR1 (Supplementary Figure S6). A possible explanation for this is that the positive selection on *tri5* occurred before it was located at GR1.

The above observations suggest that the evolutionary history of the *Trichoderma tri5* has been complex. Nevertheless, we propose a relatively simple three-step evolutionary model that accounts for the observations. Step 1 – a common ancestor of extant *Trichoderma* lineages had a *tri* cluster that included *tri5*, but subsequently lost *tri5* through gene deletion or pseudogenization. Step 2 – the *Trichoderma* ancestor reacquired *tri5 via* horizontal transfer from a fungus that occupied a markedly different habitat than other trichothecene-producing fungi for some of its evolutionary history. This is consistent with evidence for positive selection on *tri5*. Step 3 – when it was horizontally transferred, *tri5* integrated at GR1 in the ancestral *Trichoderma* genome. This model accounts for the observations noted above, and we have found precedence for each step of the model. For example, the absence of *tri4* in *Spicellum roseum* provides precedence for loss (Step 1) and subsequent reacquisition (Step 2) of an early biosynthetic step essential for formation of trichothecenes (Proctor et al., 2018). Further, there is an increasing body of evidence for horizontal transfer of secondary metabolite biosynthetic genes among fungi (Step 2; Slot and Rokas, 2011; Proctor et al., 2013; Villani et al., 2019). Although there is precedence for positive selection and horizontal transfer of secondary metabolite biosynthetic genes in fungi, we are not aware of an example of transfer of a gene that has undergone positive selection prior to the transfer. In 2014, there were publications on genome sequences for trichothecene-producing species of only two fungal genera: *Fusarium* and *Stachybotrys* (Ma et al., 2010; Semeiks et al., 2014). Today, there are publications on genome sequences of trichothecene-producing species in at least 12 genera (Pitt et al., 2017; Proctor et al., 2020; Geiser et al., 2021). As more genome sequences are generated and analyzed, they are likely to provide further insight into the evolutionary history of *tri5* and other *tri* genes in *Trichoderma*, and facilitate refinement or rejection of the model proposed above.

### Genomic Location and Functionality of *Trichoderma tri5*

Although *tri5* occurred at different genomic locations in some *Trichoderma* species, it was at the same location (GR1) in all species that had the *tri* cluster and that produced trichothecenes. The four species that had *tri5* at other genomic locations (GR2–GR5), lacked the *tri* cluster and, therefore, did not produce trichothecenes (Figure 2; Table 1). Whether this correlation is biologically significant is not known, but the correlation raises the question, is the presence of *tri5* at location GR1 more favorable for trichothecene production than its presence at other genomic locations? If the answer is yes, the occurrence of *tri5* at locations GR2–GR5 could represent a relaxation of selection for its occurrence at GR1. The *T. gamsii* T065 and *T. polysporum tri5* homologs, which occur at GR3 and GR4 respectively, differed in their ability to complement the *T. arundinaceum Δtri5* mutant. Thus, the occurrence of *tri5* at locations other than GR1 has not necessarily caused *tri5* to lose its function in trichodiene biosynthesis.

Of the nine *Trichoderma* species that have *tri5* but lack the *tri* cluster, *T.* cf. *fertile*, *T. margaretense*, and *T. rhododendri* have *tri5* homologs with 1–3 point mutations that introduce internal stop codons or a frameshift and, as a result, have potential to render the TRI5 protein nonfunctional (Supplementary Figure S10). By contrast, six of the nine species have *tri5* homologs that are apparently functional based on their DNA sequences, and further the function of the *T. decipiens*, *T. polysporum*, and *T. stromaticum tri5* homologs was demonstrated by their ability to complement the *T. arundinaceum Δtri5* mutant. The presence of a functional *tri5* homolog in some *Trichoderma* species that lack the *tri* cluster raises the question, what is the function of *tri5* in the absence of trichothecene production? Our analyses of volatiles produced by transformants of the *T. arundinaceum Δtri5* mutant carrying various *tri5* homologs failed to provide support for the hypothesis that the homologs confer the ability to produce a terpene(s) other than trichodiene. A possible alternative explanation is evident from a study in which the *T. arundinaceum tri5* was overexpressed in the biocontrol agent *T. harzianum*, which normally lacks *tri5* and the *tri* cluster (Table 1; Taylor et al., 2021). The *tri5*-expressing *T. harzianum* strain produced trichodiene and had enhanced biocontrol activity against *Fusarium graminearum*, a trichothecene-producing fungus that is a predominant cause of ear rot of maize and Fusarium head blight of wheat and barley. In addition, *tri5*-expressing *T. harzianum* volatiles, which included trichodiene, downregulated *tri* gene expression and trichothecene production in *F. graminearum* (Taylor et al., 2021). Thus, we propose the following model: in *Trichoderma* species that have a functional *tri5* but lack the *tri* cluster, trichodiene production provides a competitive advantage by inhibiting production of trichothecenes and/or other toxic terpenoids in other fungi.

### Trichothecene Biosynthesis in *Trichoderma* Species

**Figure 9:**
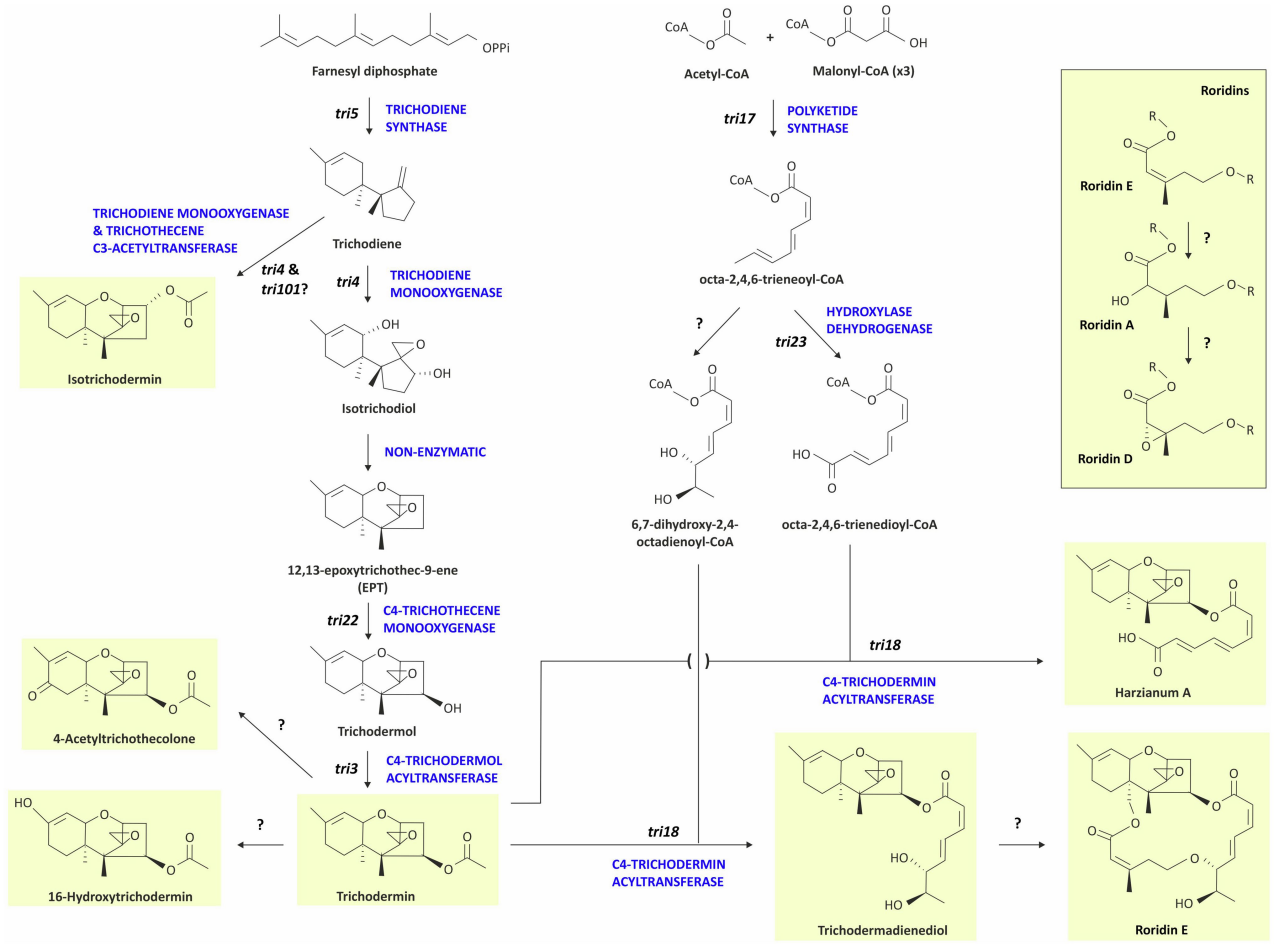
Proposed biosynthetic pathway for *Trichoderma* trichothecenes. Arrows correspond to biosynthetic steps and enzyme activities already described, except for those labeled with question marks, which point out the genes not yet identified or characterized. Chemical structures shaded in yellow correspond to the final trichothecene analogs detected in the *Trichoderma* species analyzed in the present work and included in Table 1. Genes are indicated in bold and black lowercase letters in italics, and enzymes are indicated by bold and blue uppercase letters.

Based on knowledge of the roles of the various *tri* genes in trichothecene biosynthesis, we have proposed a biosynthetic scheme to explain the biochemical relationships of trichothecene analogs that have been reported to be produced by *Trichoderma* species (Figure 9). Although trichodermin is a pathway endpoint in some species such as *T. brevicompactum* (Tijerino et al., 2011), in the proposed scheme, trichodermin is also an intermediate from which four pathway branches arise. That trichodermin is an intermediate in HA biosynthesis has been demonstrated previously (Lindo et al., 2019). Given the similarity in structure the octa-2,4,6-trienedioyl substituent of HA to the 8-carbon polyketide portion of the macrolide ring of roridins, it is possible that trichodermin is also an intermediate in roridin biosynthesis as well. With the exception of the *tri17*-encoded polyketide synthase, the genes that confer macrolide ring formation in *Trichoderma* and other fungi are not known (Semeiks et al., 2014; Proctor et al., 2018; Zhu et al., 2020). Formation of 4-acetyltrichothecolone and 16-hydroxytrichodermin from trichodermin would require only one or two biochemical reactions (Figure 9). In *Fusarium* species, oxygenation of trichothecenes at carbon atom 8 (C8) is catalyzed by a *tri1*-encoded cytochrome P450 monooxygenase. Analysis of the *T. psychrophilum* genome sequence did not reveal the presence of a *tri1* homolog. Therefore, a gene other than *tri1* must confer 8-oxygenation in *T. psychrophilum*. As far as we are aware, a *tri* gene that confers 16-hydroxylation has not been identified in any trichothecene-producing fungus. Thus, the gene required for formation of 16-hydroxy substituent of 16-hydroxytrichodermin produced by *T. albolutescens* remains to be determined (Ryu et al., 2017).

In the biosynthetic scheme, we proposed that the formation of isotrichodermin occurs *via* 3-hydroxylation of trichodiene rather than 3-hydroxylation of EPT because the latter modification occurs *via* the activity of TRI4 during trichothecene biosynthesis in *Fusarium* species. That is, *Fusarium* TRI4 homologs catalyze hydroxylation of trichodiene at C2, C3, C11, and C13, which causes a nonenzymatic cyclization resulting in the formation of isotrichodermol (3-hydroxy EPT; McCormick et al., 2006; Tokai et al., 2007). The *tri101-*encoded acetyltransferase then catalyzes acetylation of the 3-hydroxy substituent to form isotrichodermin (3-acetyl EPT; McCormick et al., 1999). The activity of TRI4 marks a fundamental difference in trichothecene biosynthesis in *Trichoderma* and *Fusarium*. Analysis of *T. arundinaceum tri4* indicates that the *Trichoderma* TRI4 catalyzes hydroxylation of trichodiene at only C2, C11, and C13, resulting information of EPT rather than 3-hydroxy EPT (Cardoza et al., 2015). This activity is consistent with the absence of a 3-oxygen atom in all reported *Trichoderma* trichothecenes except for isotrichodermin produced by *T. albolutescens*. It is possible, however, that the *T. albolutescens* TRI4 homolog has low levels of trichodiene 3-hydroxylation activity, as is the case for the *Myrothecium roridum* TRI4 (McCormick and Alexander, 2007). Further, because many fungi have trichothecene 3-acetylation activity (Khatibi et al., 2011), it would not be unusual for *T. albolutescens* to also have such activity. Thus, we propose that production of isotrichodermin (3-acetyl EPT) by *T. albolutescens* results from 3-hydroxylation activity of its TRI4 homolog and a 3-acetylation activity that is relatively common among fungi.

### Conclusion

The results of this study expand the understanding of trichothecene production in filamentous fungi by demonstrating that production is discontinuously distributed over a wide breadth of phylogenetic diversity of the genus *Trichoderma*. This distribution of trichothecene production among *Trichoderma* species suggests the toxins could play a role in the ability of some species to survive and compete under certain environmental conditions. The finding that functional homologs of *tri5* are retained in some species suggests that trichodiene production in the absence of trichothecene production could also provide an ecological advantage. This is turn consistent with the hypothesis proposed by Taylor et al. (2021) that trichodiene can act as a signaling molecule. Further, the genome sequences of *Trichoderma* species that produce unusual trichothecene analogs, such as 4-acetyltrichothecolone and 16-hydroxytrichodermin, should provide a source to identify novel trichothecene biosynthetic genes that could provide insight into the ecological roles of trichothecenes in this important genus of microorganisms.

## Data Availability Statement

The datasets presented in this study can be found in online repositories. The names of the repository/repositories and accession number(s) can be found in the article/Supplementary Material.

## Author Contributions

SG and RP conceived the work, designed the experiments, performed genome sequencing, participated in bioinformatics analyses, and coordinated the work of the rest of the coauthors. SM, RC, H-SK, MB, LY, MV, PC, and GC-H performed the chemical studies, fungal transformations, tri5 mutant isolation and complementation, and antifungal experiments, and participated in the bioinformatics analyses. LS participated in the bioinformatics analyses. WJ provided fungal material. W-YZ and CW participated in the fungal genome sequencing. All authors collaborated in the discussion of the results, and contributed to the writing and to the corrections made to reach the final version of this manuscript.

## Funding

This work is a part of the Spanish I+D+i Grant RTI2018-099600-B-I00, financed by the MCIN/AEI/10.13039/501100011033. This work was supported in part by the U.S. Department of Agriculture, Agricultural Research Service.

## Author Disclaimer

Mention of trade names or commercial products in this publication is solely for the purpose of providing specific information and does not imply recommendation or endorsement by the U.S. Department of Agriculture. USDA is an equal opportunity provider and employer.

## Conflict of Interest

The authors declare that the research was conducted in the absence of any commercial or financial relationships that could be construed as a potential conflict of interest.

## Publisher’s Note

All claims expressed in this article are solely those of the authors and do not necessarily represent those of their affiliated organizations, or those of the publisher, the editors and the reviewers. Any product that may be evaluated in this article, or claim that may be made by its manufacturer, is not guaranteed or endorsed by the publisher.
