# Supplementary material for: Distribution, Function, and Evolution of a Gene Essential for Trichothecene Toxin Biosynthesis in Trichoderma
Source: Front Microbiol. 2021 Dec 2;12:791641. doi: 10.3389/fmicb.2021.791641 (PMC8675399; doi:10.3389/fmicb.2021.791641)
Supplement: Supplementary file 1 [file Data_Sheet_1.pdf]

Table S1a. Genomic sequences of *Trichoderma* spp. that were retrieved from the National Center for Biotechnology Information (NCBI) database for their use in the phylogenetic analyses carried out in the present work.

Species	GenBank accession number	Reference
Trichoderma asperellum CBS 433.97	GCF_003025105.1	Druzhinina, I.S., Chenthamara, K., Zhang, J., Atanasova, L., Yang, D., Miao, Y., Rahimi, M.J., Grujic, M., Cai, F., Pourmehdi, S., Salim, K.A., Pretzer, C., Kopchinskiy, A.G., Henrissat, B., Kuo, A., Hundley, H., Wang, M., Aerts, A., Salamov, A., Lipzen, A., LaButti, K., Barry, K., Grigoriev, I.V., Shen, Q., Kubicek, C.P. 2018. Massive lateral transfer of genes encoding plant cell wall-degrading enzymes to the mycoparasitic fungus *Trichoderma* from its plant-associated hosts. *PLoS Genet*. 14: e1007322. http://doi.org/10.1371/journal.pgen.1007322.
Trichoderma citrinoviride TUCIM 6016	GCF_003025115.1	
Trichoderma guizhouense NJAU 4742	GCA_002022785.1	
Trichoderma harzianum CBS 226.95	GCF_003025095.1	
Trichoderma atroviride IMI 206040	GCF_000171015.1	Kubicek CP, Herrera-Estrella A, Seidl-Seiboth V, et al. 2011. Comparative genome sequence analysis underscores mycoparasitism as the ancestral life style of *Trichoderma*. *Genome Biol*. 12(4): R40. http://doi.org/10.1186/gb-2011-12-4-r40
Trichoderma virens Gv29-8	GCF_000170995.1	
Trichoderma hamatum GD12	GCA_000331835.2	Studholme DJ, Harris B, Le Cocq K, Winsbury R, Perera V, Ryder L, Ward JL, Beale MH, Thornton CR, Grant M. 2013. Investigating the beneficial traits of *Trichoderma hamatum* GD12 for sustainable agriculture-insights from genomics. *Front. Plant Sci.* 4: 258. http://doi.org/10.3389/fpls.2013.00258.
Trichoderma arundinaceum IBT 40837	GCA_003012105.1	Proctor, R.H., McCormick, S.P., Kim, H.S., Cardoza, R.E., Stanley, A.M., Lindo, L., Kelly, A., Brown, D.W., Lee, T., Vaughan, M.M., Alexander, N.J., Busman, M., Gutiérrez, S. 2018. Evolution of structural diversity of trichothecenes, a family of toxins produced by plant pathogenic and entomopathogenic fungi. *PLoS Pathog*. 14(4): e1006946. http://doi.org/10.1371/journal.ppat.1006946
Trichoderma brevicompactum IBT 40841	GCA_003012085.1	
Trichoderma atrobrunneum ITEM 908	GCA_003439915.1	Fanelli, F., Liuzzi, V.C., Logrieco, A.F., Altomare, C. 2018. Genomic characterization of *Trichoderma atrobrunneum* (*T. harzianum* species complex) ITEM 908: insight into the genetic endowment of a multi-target biocontrol strain. *BMC Genomics*. 19: 662. http://doi.org/10.1186/s12864-018-5049-3
Trichoderma koningii JCM 1883	GCA_001950475.1	Manabe, R., Endoh, R., Uzuhashi, S., Okada, G., Takashima, M., Ohkuma, M. 2016. *Trichoderma koningii* strain JCM 1883, whole genome shotgun sequencing project. Direct submission
Trichoderma koningiopsis P0S7	GCA_002246995.1	Castrillo, M.L., Bich, G.A., Modenutti, C.P., Turjanski, A.G., Zapata, P.D., Villalba, L. 2017. First whole-genome shotgun sequence of a promising cellulase secretor, *Trichoderma koningiopsis* strain POS7. Genome Announc. 5: e00823-17. http://doi.org/10.1128/genomeA.00823-17
Trichoderma lentiforme CFAM-422	GCA_011066345.1	Steindorff, A.S., Formighieri, E.F., Midorikawa, G.E.O., Tamietti, M.S., Ramos, E.Z., Silva, A.S., Bon, E.P.S., Mendes, T.D., Damaso, M.C.T., Favaro, L.C.L. 2018. *Trichoderrma lentiforme* strain CFAM-422, whole genome shotgun sequence project. Direct submission.

*Trichoderma oligosporum C*GMCC 3.17527	GCA_015266385.1	Wang, C., Zeng, Z., Zhuang, w. 2020. Comparative molecular evolution of chitinases in Ascomycota with emphasis on mycoparasitism lifestyle. Direct submission.
Trichoderma parareesei CBS125925	GCA_001050175.1	Yang, D., Pomraning, K., Kopchinskiy, A., Karimi-Aghcheh, R., Atanasova, L., Chenthamara, K., Baker, S.E., Zhang, R., Shen, Q., Freitag, M., Kubicek, C.P., Druzhinina, I.S. 2017. Genome sequence and annotation of *Trichoderma parareese*i, the ancestor of the cellulase producer *Trichoderma reesei*. *Genome Announc.* 20153(4). http://doi.org/10.1128/genomeA.00885-15
Trichoderma reesei QM6a	GCF_000167675.1	Li, W.C., Huang, C.H., Chen, C.L., Chuang, Y.C., Tung, S.Y., Wang, T.F. 2017.*Trichoderma reesei* complete genome sequence, repeat-induced point mutation, and partitioning of CAZyme gene clusters. *Biotechnol Biofuels.* 10: 170. http://doi.org/10.1186/s13068-017-0825-x

Table S1b. Genomic sequences retrieved from the National Center for Biotechnology Information (NCBI) database of non-*Trichoderma* species for their use in the phylogenetic analyses carried out in the present work.

Species	GenBank accession number	Reference
Fusarium longipes NRRL 20695	PXOG00000000	Proctor, R.H., McCormick, S.P., Kim, H.S., Cardoza, R.E., Stanley, A.M., Lindo, L., Kelly, A., Brown, D.W., Lee, T., Vaughan, M.M., Alexander, N.J., Busman, M., Gutiérrez, S. 2018. Evolution of structural diversity of trichothecenes, a family of toxins produced by plant pathogenic and entomopathogenic fungi. *PLoS Pathog*. 14 (4): e1006946. http://doi.org/10.1371/journal.ppat.1006946
Fusarium fasciculatum NRRL 13405	PXXK00000000	
Fusarium sporotrichioides NRRL 3299	PXOF00000000	
Microcyclospora tardicrescens HJS 1936	PXOE00000000	
Myrothecium roridum NRRL 2183	PXOD00000000	
Spicellum ovalisporum DAOM 186447	PXOC00000000	
Trichothecium roseum DAOM 195227	PXNY00000000	
Aspergillus hancockii	GCA_001696595.2	Pitt, I.P., Lange, L., Lacey, A.E., Vuong, D., Midgley, D.J., Greenfied, P., Bradbury, M.I., Lacey, E., Busk, P.K., Pilgaard, B.,, Chooi, Y.-H., Piggott, A.M. 2017. *Aspergillus hancockii* sp. nov., a biosynthetically talented fungus endemic to southeastern Australia soils. PLoS ONE 12: 30170254. https://doi.org/10.1371/journal.pone.0170254
Beauveria bassiana ARSEF 2860	ADAH00000000	Xiao, G., Ying, S.-H., Zheng, P., Wang, A.-L., Zhang, S., Xie, S.-Q., Shang, Y., Leger, R.J., Zhao, G.-P., Wang, C., Geng, M.-G. 2012. Genomic perspectives on the evolution of fungal entomopathogenicity in *Beauveria bassiana*. Sci. Rep. 2: 483. http://doi.org/10.1038/srep00483.
Cordyceps confragosa UM487	GCA_001653215.1	Fine-scale spatial genetic structure of a fungal parasite of coffee scale insects: Unpublished – Jackson,D., Zemenick,K.A., Malloure,B., Quandt,C.A., James,T.Y.
Fusarium graminearum PH-1	AACM00000000	Walkowiak, S., Rowland, O., Rodrigue, N., Subramaniam, R. 2016. Whole genome sequencing and comparative genomics of closely related Fusarium Head Blight fungi: *Fusarium graminearum, F. meridionale* and *F. asiaticum*. BMC Genomics. 17: 1014. http://doi.org/10.1186/s12864-016-3371-1
Stachybotrys chartarum 40293	ASEQ00000000	Semeiks, J., Borek, D., Otwinowski, Z., Grishin, N.V. 2014. Comparative genome sequencing reveals chemotype-specific gene clusters in the toxigenic black mold *Stachybotrys*. 15: 590. https://doi.org/10.1186/1471-2164-15-590
Stachybotrys chlorohalonata IBT 40285	APWP00000000	

Table S2. Housekeeping genes used for the phylogenetic and evolutive analyses

Gene	*T. arundinaceum* Gene Model	Predicted Protein Product
cpr1	TARUN_9366	Cytochrome P450 Reductase
dpa1	TARUN_6778	DNA Polymerase Alpha Subunit
dpd1	TARUN_237	DNA Polymerase Delta Subunit
erg1	TARUN_9990	Squalene Monooxygenase
fas1	TARUN_2912	Fatty Acid Synthase Alpha Subunit
fas2	TARUN_2913	Fatty Acid Synthase Beta Subunit
his3	TARUN_10324	Histone H3
lae1	TARUN_10114	Global Regulatory Gene/Chromatin Methyl Transferase
lcb1	TARUN_6733	Sphinganine Palmitoyl Transferase Subunit 1
lcb2	TARUN_696	Sphinganine Palmitoyl Transferase Subunit 2
mcm7	TARUN_2249	DNA Replication Licensing Factor
pgk1	TARUN_275	Phosphoglycerate Kinase
rpb1	TARUN_7976	RNA Polymerase II Largest Subunit
rpb2	TARUN_7706	RNA Polymerase II Subunit 2
sph1	TARUN_9560	Longevity Assurance Factor/Sphinganine N Acyl Transferase Subunit 1
top1	TARUN_773	Topoisomerase
tps1	TARUN_4479	Trehalose Phosphate Synthase
tsr1	TARUN_1474	Ribosomal Biogenesis Protein
tub1	TARUN_4212	Tubulin Alpha Subunit
ubt1	TARUN_8065	Ubiquitin Thiolesterase

Note. Genes in red color are those used to construct the phylogenetic tree including *Trichoderma* and other fungi belonging to the order Hypocreales that were previously shown to have a *tri5* copy in their genomes (Proctor et al., 2018).

Proctor, R.H., McCormick, S.P., Kim, H.-S., Cardoza, R.E., Stanley, A.M., Lindo, L., Kelly, A., Brown, D.W., Lee, T., Vaughan, M.M., Alexander, N.J., Busman, M., Gutiérrez. S. (2018). Evolution of structural diversity of trichothecenes, a family of toxins produced by plant pathogenic and entomopathogenic fungi. *PloS Pathog.* 14(4)e1006946. doi: 10.1371/journal. ppat.1006946

Table S3. Oligonucleotides used

Species	**Oligo name**	**Sequence 5´-3´**	
**Oligonucleotides to construct plasmid pΔtri5**			
T. arundinaceum	tri5_5rF_BamHI	GGATCCCTATTGAACCGTGGCAAA	
	tri5_5Rr_SmaI	CCCGGGGCTCAACCATGATGATGA	
	tri5_3rF_SmaI	CCCGGGGCTTATTCGAGGCTATG	
	tri5_3rR_SalI	GTCGACAGGCTCTGGAATAGCATA	
**Oligonucleotides to analyze *T. arundinaceum tri5* deleted mutants**			
T. arundinaceum	T5_5rr	CTGTGATGCAGTAAGGCT	
	T5_3rr	GCATGTGATACGCATGCA	
	PgpdA-d	ATCATCCACTGCACCTCA	
	TrpC-d	GTAACCATGCATGGTTGC	
**Oligonucleotides to amplify *tri5* gene from non-trichothecene producers *Trichoderma* species**			
T. arundinaceum	TARUN_T5_5	GTTGAGCTCAACGACGTC	
	TARUN_T5_3	CTACTGGGCGACGGAGGA	
T. stromaticum	TSTRO_T5_5	GTTGACCTCAACAGCATT	
	TSTRO_T5_3	TTAGCCATCCCTCTCTTG	
T. cf. *fertile*	TFERT_T5_5	GTTGAGCTCAACAACGCT	
	TFERT_T5_3	TCATTGTTCCAGCCTCTT	
T. polysporum	TPOLY_T5_5	GTTGAGCTTAATGATATC	
	TPOLY_T5_3	TCACGCAAAGGATAAGAT	
T. decipiens	TDECI_T5_5	GATGGGATCAATATTGTC	
	TDECI_T5_3	TCAGGGTTTCCCAGCAAG	
T. gamsii T065	TGAMS_T5_5	GTTGAGCTCAATGATGTT	
	TGAMS_T5_3	TCACCACTCAACAGACGT	
T. balearicum	TBALE_T5_5	CCTGAGCTCAATGACGTC	
	TBALE_T5_3	TCAGTACTGAGCAACAGA	
**Oligonucleotides for qPCR analysis**			**PCR efficiency (%)**
***tri5* gene**			
T. arundinaceum	TARUN_tri5_F	TGTGTCGGCTCTTCAATCTG	91.3
	TARUN_tri5_R	GCTTATCGAGAGCCTGATCG	
T. stromaticum	TSTRO_T5_L1	GCAGTTCCCTCGAACTACCT	103.5
	TSTRO_T5_R1	GCTCGGCAGCTTTCGAATAA	
T. cf. *fertile*	TFERT_T5_L1	TGTCGTGTCTCCAAGGAAGT	108.0
	TFERT_T5_R1	CGTACCAGGTCGCCATACTA	
T. polysporum	TPOLY_T5_L1	GCGTTTATTCCTGGTGCCAT	107.9
	TPOLY_T5_R1	CCATGGTATCAGCAGGCTCT	
T. decipiens	TDECI_T5_L1	CTGGCTGCTGCTGTTAAGAC	107.8
	TDECI_T5_R1	GCGGTCTGGCATTTAGAAGG	
T. gamsii T065	TGAMS_T5_L1	AAAGTGCCTCCTAAGCGTCT	106.3
	TGAMS_T5_R1	GTATCGGCAGGCTCTTCTCT	
T. balearicum	TBALE_T5_L1	CGCTAGTGACTATCCCGGTT	110.6
	TBALE_T5_R1	GAGCAATGGCTGTGGTGATT	
**actin gene**			

T. arundinaceum	TARUN_actin_F	ACTGGGACGACATGGAGAAG	92.1
	TARUN_actin_R	GGCCTGGATGGAGACATAGA	
T. stromaticum	TSTRO_actin_L1	TGAGCGTGGTTACACCTTCT	106.6
	TSTRO_actin_R1	GCCAATGGTGATGACCTGAC	
T. cf. *fertile*	TFERT_actin_L1	AGGAGAAGCTCTGCTACGTC	94.9
	TFERT_actin_R1	AAGGACAGAAGGCTGGAACA	
T. polysporum	TPOLY_actin_L1	AGGAGAAGCTCTGCTACGTC	105.9
	TPOLY_actin_R1	AAGGACAGAAGGCTGGAACA	
T. decipiens	TDECI_actin_L1	TCCAGGCTGTTCTGTCTCTG	108.0
	TDECI_actin_R1	CGACCAGCCATATCAACACG	
T. gamsii T065	TGAMS_actin_L1	AGGAGAAGCTCTGCTACGTC	106.8
	TGAMS_actin_R1	ACCGCTCTCAAGACCAAGAA	
T. balearicum	TBALE_actin_L1	TGACCCAGATCGTCTTCGAG	107.4
	TBALE_actin_R1	GTCAACACGAGCAATAGCGT	

Table S4a. GenBank database (National Center for Biotechnology Information) accession numbers of housekeeping gene sequences used for the phylogenetic studies, which were retrieved from the genome sequences generated as part of the current study.

	**Gene/GenBank accession number**									
Species	***cpr1***	***dpa1***	***dpd1***	***erg1***	***fas1***	***fas2***	***his3***	***lae1***	***lcb1***	***lcb2***
T. albolutescens	OK513278	OK626232	OL335883	OK659798	OK659817	OK659836	OL101211	OL101268	OL101230	OL101249
T. aurantioeffusum	OK513279	OK626251	OL335884	OK659799	OK659818	OK659837	OL101212	OL101269	OL101231	OL101250
T. balearicum	OK513280	OK626233	OL335885	OK659800	OK659819	OK659838	OL101213	OL101270	OL101232	OL101251
T. calamagrostidis	OK392595	OK626234	OL335886	OK659801	OK659820	OK659839	OL101214	OL101271	OL101233	OL101252
T. crystalligenum	OK513281	OK626235	OL335887	OK659802	OK659821	OK659840	OL101215	OL101272	OL101234	OL101253
T. decipiens	OK513282	OK626236	OL335888	OK659803	OK659822	OK659841	OL101216	OL101273	OL101235	OL101254
T. deliquescens	OK513283	OK626237	OL335889	OK659804	OK659823	OK659842	OL101217	OL101274	OL101236	OL101255
T.* cf. *fertile	OK513284	OK626238	OL335890	OK659805	OK659824	OK659843	OL101218	OL101275	OL101237	OL101256
***T. gamsii* T065**	OK513285	OK626239	OL335891	OK659806	OK659825	OK659844	OL101219	OL101276	OL101238	OL101257
T. margaretense	OK513286	OK626240	OL335892	OK659807	OK659826	OK659845	OL101220	OL101277	OL101239	OL101258
T. oligosporum	OK513277	OK626241	OL355103	OL343670	OL343671	OL343672	OL343673	OL343674	OL343675	OL343676
T. polysporum	OK513287	OK626242	OL335893	OK659808	OK659827	OK659846	OL101221	OL101278	OL101240	OL101259
T. protrudens	OK513288	OK626243	OL335894	OK659809	OK659828	OK659847	OL101222	OL101279	OL101241	OL101260
T. psychrophillum	OK513289	OK626244	OL335895	OK659810	OK659829	OK659848	OL101223	OL101280	OL101242	OL101261
T. rhododendri	OK513290	OK626245	OL335896	OK659811	OK659830	OK659849	OL101224	OL101281	OL101243	OL101262
T. rodmanii	OK513291	OK626246	OL335897	OK659812	OK659831	OK659850	OL101225	OL101282	OL101244	OL101263
T. rubi	OK513292	OK626247	OL335898	OK659813	OK659832	OK659851	OL101226	OL101283	OL101245	OL101264
T. stromaticum	OK513294	OK626248	OL335899	OK659814	OK659833	OK659852	OL101227	OL101284	OL101246	OL101265
T. taxi	OK413293	OK626249	OL335900	OK659815	OK659834	OK659853	OL101228	OL101285	OL101247	OL101266
T. turrialbense	OK513295	OK626250	OL335901	OK659816	OK659835	OK659854	OL101229	OL101286	OL101248	OL101267

	**Gene/GeneBank accession number**									
Species	***mcm7***	***pgk1***	***rpb1***	***rpb2***	***sph1***	***top1***	***tps1***	***tsr1***	***tub1***	***ubt1***
T. albolutescens	OK813947	OK813928	OK813909	OK813890	OK656756	OK656737	OK656718	OK631850	OK587421	OK587402
T. aurantioeffusum	OK813948	OK813929	OK813910	OK813891	OK656757	OK656738	OK656719	OK631851	OK587422	OK587403
T. balearicum	OK813949	OK813930	OK813911	OK813892	OK656758	OK656739	OK656720	OK631852	OK587423	OK587404
T. calamagrostidis	OK813950	OK813931	OK813912	OK813893	OK656759	OK656740	OK656721	OK631853	OK587424	OK587405
T. crystalligenum	OK813951	OK813932	OK813913	OK813894	OK656760	OK656741	OK656722	OK631854	OK587425	OK587406
T. decipiens	OK813952	OK813933	OK813914	OK813895	OK656761	OK656742	OK656723	OK631855	OK587426	OK587407

T. deliquescens	OK813953	OK813934	OK813915	OK813896	OK656762	OK656743	OK656724	OK631856	OK587427	OK587408
T.* cf. *fertile	OK813954	OK813935	OK813916	OK813897	OK656763	OK656744	OK656725	OK631857	OK587428	OK587409
***T. gamsii* T065**	OK813955	OK813936	OK813917	OK813898	OK656764	OK656745	OK656726	OK631858	OK587429	OK587410
T. margaretense	OK813956	OK813937	OK813918	OK813899	OK656765	OK656746	OK656727	OK631859	OK587430	OK587411
T. oligosporum	OL335905	OL335906	OL335907	OL335908	OL335902	OL335903	OL335904	OK631849	OK587440	OK504622
T. polysporum	OK813957	OK813938	OK813919	OK813900	OK656766	OK656747	OK656728	OK631860	OK587431	OK587412
T. protrudens	OK813958	OK813939	OK813920	OK813901	OK656767	OK656748	OK656729	OK631861	OK587432	OK587413
T. psychrophillum	OK813959	OK813940	OK813921	OK813902	OK656768	OK656749	OK656730	OK631862	OK587433	OK587414
T. rhododendri	OK813960	OK813941	OK813922	OK813903	OK656769	OK656750	OK656731	OK631863	OK587434	OK587415
T. rodmanii	OK813961	OK813942	OK813923	OK813904	OK656770	OK656751	OK656732	OK631864	OK587435	OK587416
T. rubi	OK813962	OK813943	OK813924	OK813905	OK656771	OK656752	OK656733	OK631865	OK587436	OK587417
T. stromaticum	OK813963	OK813944	OK813925	OK813906	OK656772	OK656753	OK656734	OK631866	OK587437	OK587418
T. taxi	OK813964	OK813945	OK813926	OK813907	OK656773	OK656754	OK656735	OK631867	OK587438	OK587419
T. turrialbense	OK813965	OK813946	OK813927	OK813908	OK656774	OK656755	OK656736	OK631868	OK587439	OK587420

Table S4b. GenBank database (National Center for Biotechnology Information) accession numbers of sequences generated as part of the current study, corresponding to *tri5*- and *tri5*-related regions.

Species	**Region***	**Accession number**
T. turrialbense	*tri5* region	MZ926819
T. protrudens	*tri5* region	MZ926820
T. margaretense	*tri5* region	MZ926821
T. rodmanii	*tri5* region	MZ968859
T. aurantioeffusum	*tri5* region	MZ968994
T. gamsii T065	dbp3 region**	OK037079
	tri5 region	OK037075
	urea carboxylase region	OK037076
T. polysporum	dbp3 region**	OK094918
	tri5 region	OK094919
	urea carboxylase region	OK094920
T. rubi	*tri5* region	OK043671
T. taxi	*tri5* region	MZ825154
T. balearicum	*tri5* region	OK037078
T. crystalligenum	*tri5* region	OK094924
T. oligosporum	*tri5* region	OK094926
T. calamagrostidis	*tri5* region	MZ869853
T. psychrophilum	*tri5* region	OK094925
T. rhododendri	*tri5* region	OK094923
T. decipiens	*tri5* region	OK043672
T. deliquescens	dbp3 region	OK094921
T. albolutescens	*tri5* region	OK037077
T. cf. *fertile*	*tri5* region	OK094922
T. stromaticum	dbp3 region**	OK298475
	tri5 region	OK298474

*Note that the genomic regions whose sequences were submitted to the GenBank corresponded to those illustrated in Fig. 2.
** dbp3 region- region containing the ATP-dependent RNA helicase encoding gene, corresponding to the genomic region where *tri5* gene is located in those *Trichoderma* trichothecene producer strains (=GR1 in Fig 2).

Table S5. RNAseq analysis of expression of genes located in the *tri5* genomic region of *T. arundinaceum**.

Gene number**	Blastp highest similarity	TPM TARUN vs TPM Δ*tri6* 24h	Correspondence with genes in Fig. 2**	TPM TARUN 24h	TPM Δ*tri6* 24h
TARUN_2540	ATP-dependent RNA helicase	0.88	Red arrow	23.7	26.87
TARUN_2541	oligosaccharydyl-lipid flippase	0.94	Pink arrow	29.9	31.89
**TARUN_2542 (*tri5*)**	**Trichodiene synthase**	**53.14**	**Green arrow**	**53.14**	**1**
TARUN_2543	Cytochrome P450 monooxygenase	1.92	Yellow arrow	17.9	9.3
TARUN_2544	AraC-type transcriptional regulator	1.00	Blue arrow	1	1
TARUN_2545	Fucose-specific lectin	0.24	5	2.26	9.4
TARUN_2546	Homoserine acetyltransferase	4.26	Purple arrow	4.26	1
TARUN_2547	WD40-repeat-containing domain protein	0.80	4	105.36	132.34
TARUN_2548	Major facilitator superfamily (MFS)	0.40	Orange arrow	0.4	1
TARUN_2549	duf1446 domain-containing protein	0.64	3	1.53	2.39
TARUN_2550	Chromo domain-containing protein	0.68	2	28.96	42.48
TARUN_2551	Peptidase m20 domain protein	2.57	1	8.06	3.14
TARUN_5502***	Actin beta gamma 1	0.99	-	660.77	663.62

* Gene expression values were determined by comparison of expression levels found in *T. arundinaceum* wild type (TARUN) *versus* those found in the Δ*tri6.66*, which is a mutant deleted in the *tri6* gene that encodes a positive regulator of genes involved in trichothecene biosynthesis (Lindo et al., 2018).
**Genes included in this table correspond with those illustrated in Fig. 2 for the *tri5* genomic region of *T. arundinaceum*.
***Values for Actin beta gamma 1 encoding gene were shaded in yellow, since they were used to normalize the gene expression data, but this gene is not located in the *tri5* genomic region.
TPM= Transcripts per million of reads

Lindo, L., McCormick, S.P., Cardoza, R.E., Brown, D.W., Kim, H.-S., Alexander, N.J., Proctor, R.H., Gutiérrez, S. (2018). Effect of deletion of a trichothecene toxin regulatory gene on the secondary metabolism transcriptome of the saprotrophic fungus *Trichoderma arundinaceum*. *Fungal Genet. Biol*. 119, 29-46. doi: 10-1016/j.fgb.2018.08.002

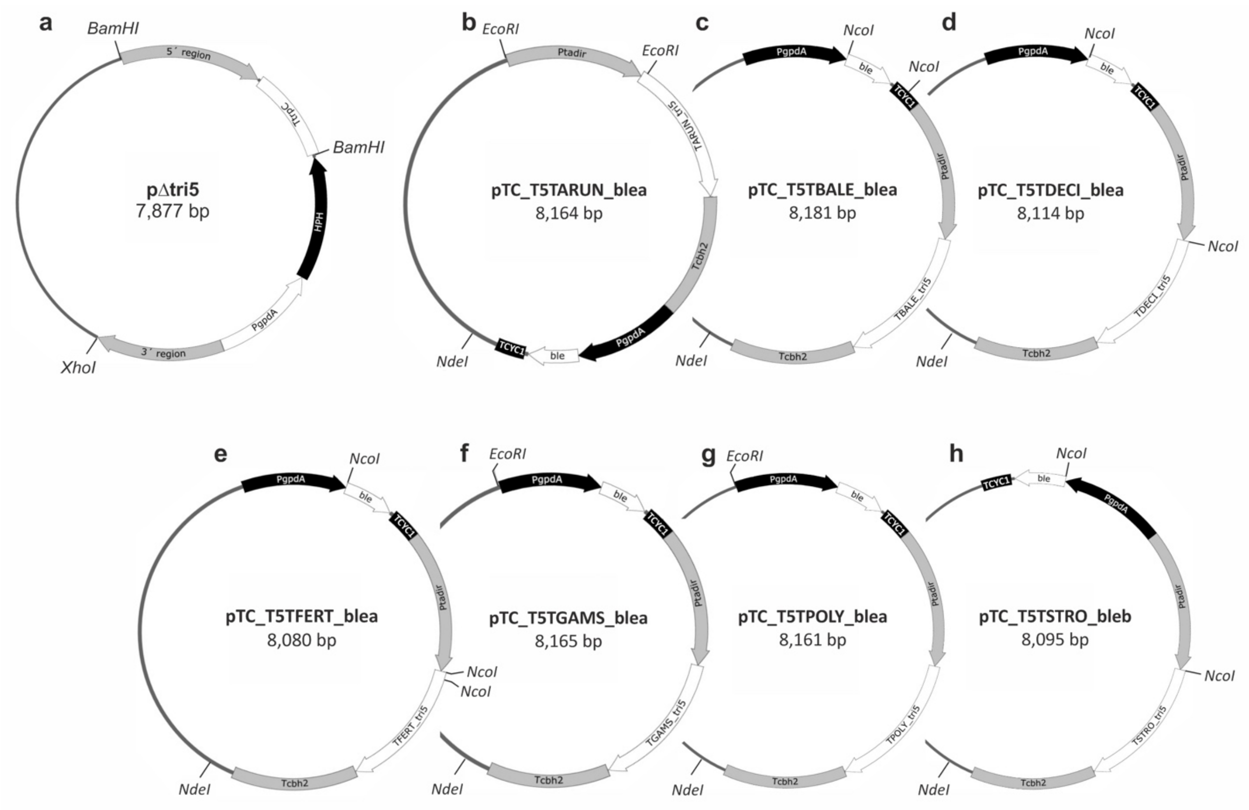

Figure S1. Plasmids used for *T. arundinaceum tri5* deletion (pΔtri5) or for *tri5* gene overexpression (pTC_T5#) studies. TARUN= *T. arundinaceum*, TBALE= *T. balearicum*, TDECI= *T. decipiens*, TFERT= *T.* cf. *fertile*, TGAMS= *T. gamsii* T065, TPOLY= *T. polysporum*, TSTRO= *T. stromaticum*.

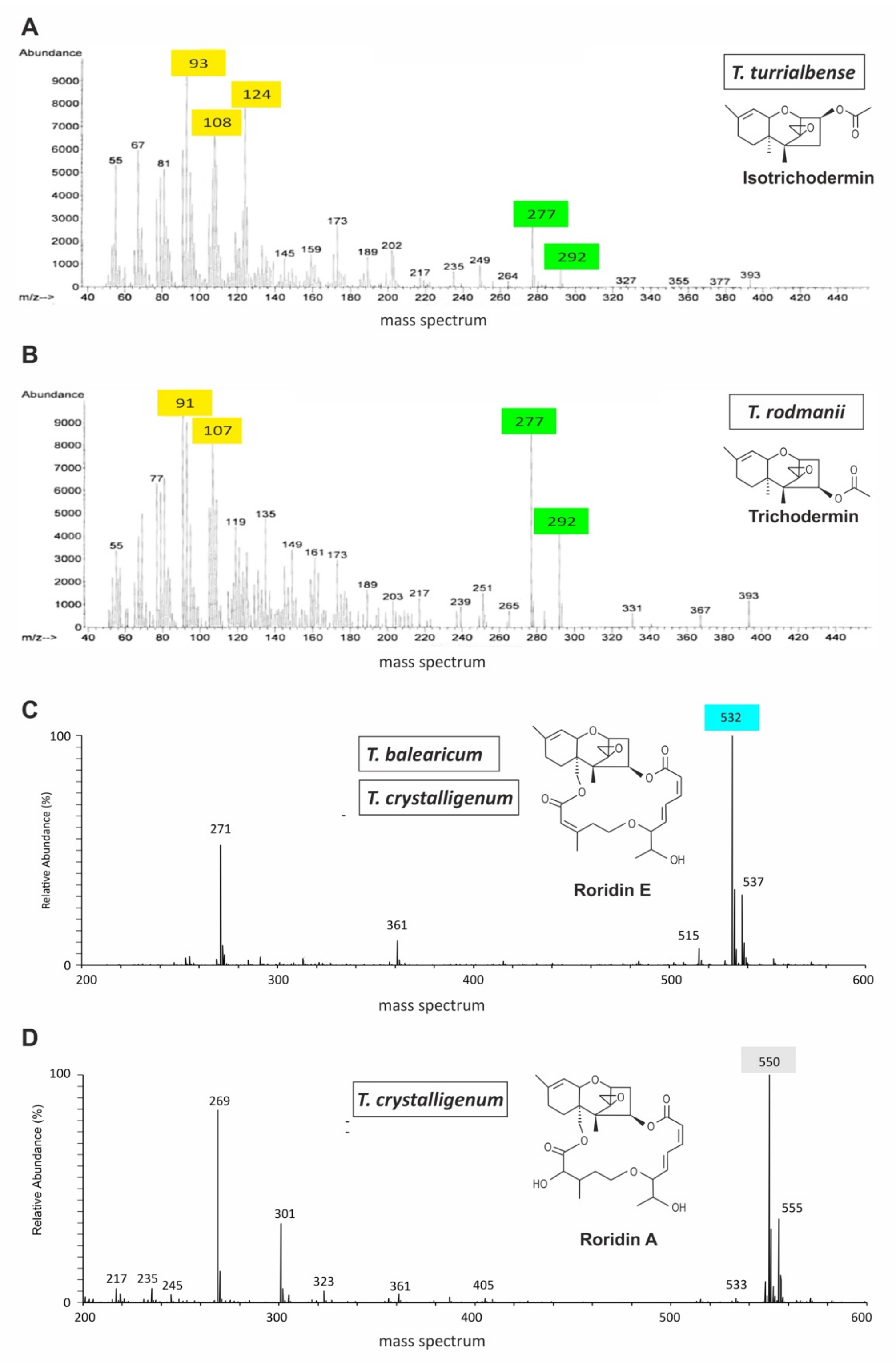

Figure S2. Total mass spectra of isotrichodermin (**A**), trichodermin (**B**); roridin E (**C**), and roridin A (**D**). Green rectangles in panels A and B correspond to common m/z peaks to isotrichodermin and trichodermin, while yellow rectangles indicated the differential m/z peaks between these two compounds. Blue and gray rectangles in panels C and D correspond to 532 and 550 ions for roridin E and roridin A, respectively, with an ammonium adduct.

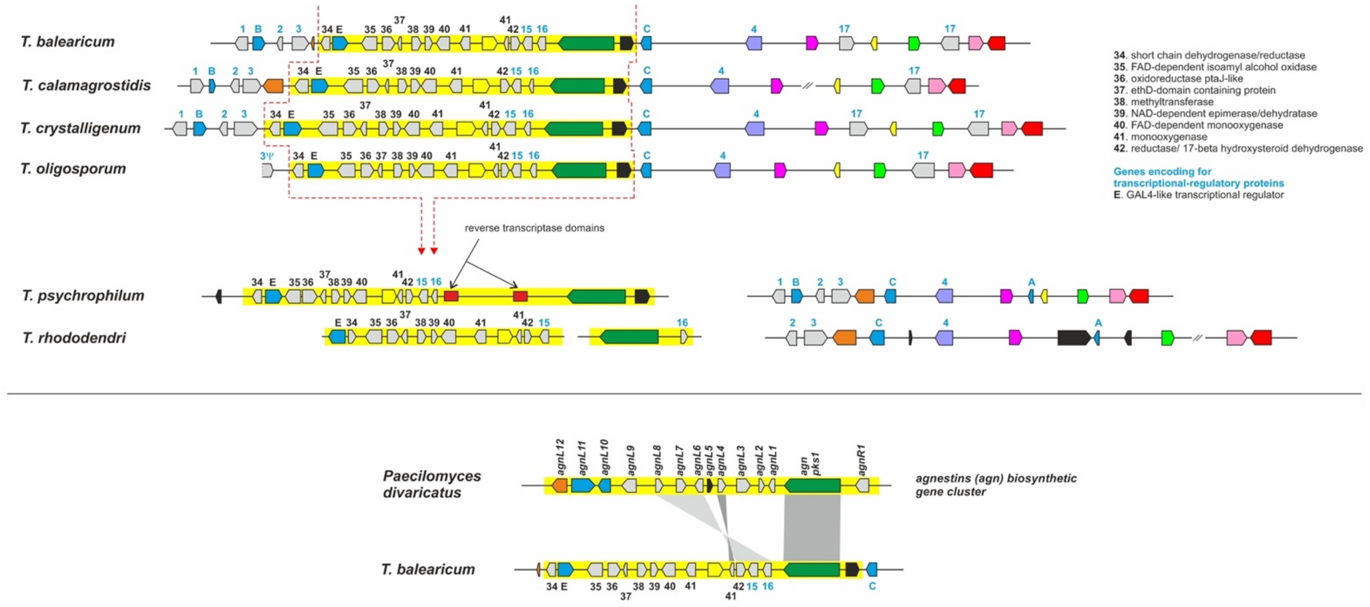

Figure S3. **Upper panel**. Analysis of genes found in the *tri5* genomic region in species of the *Psychrophila* lineage. Those genes shaded in yellow were only found in this clade among those *Trichoderma* clades included in the present study, among all the 35 species analyzed in the present work, and shown a significant degree of synteny with the *Paecylomyces divaricatus* agnestins biosynthetic cluster (**Lower panel**) (Szwalbe et al., 2018).

Szwalbe, A.J., Williams, K., Song, Z., de Mattos-Shipley, K., Vincent, J.L., Bailey, A.M., Willis, C.L., Cox, R.J., Simpson, T.J. (2018). Characterisation of the biosynthetic pathway to agnestins A and B reveals the reductive route to chrysophanol in fungi. *Chem. Sci.* 10, 233-238. doi: 10.1030/cBsc03778g

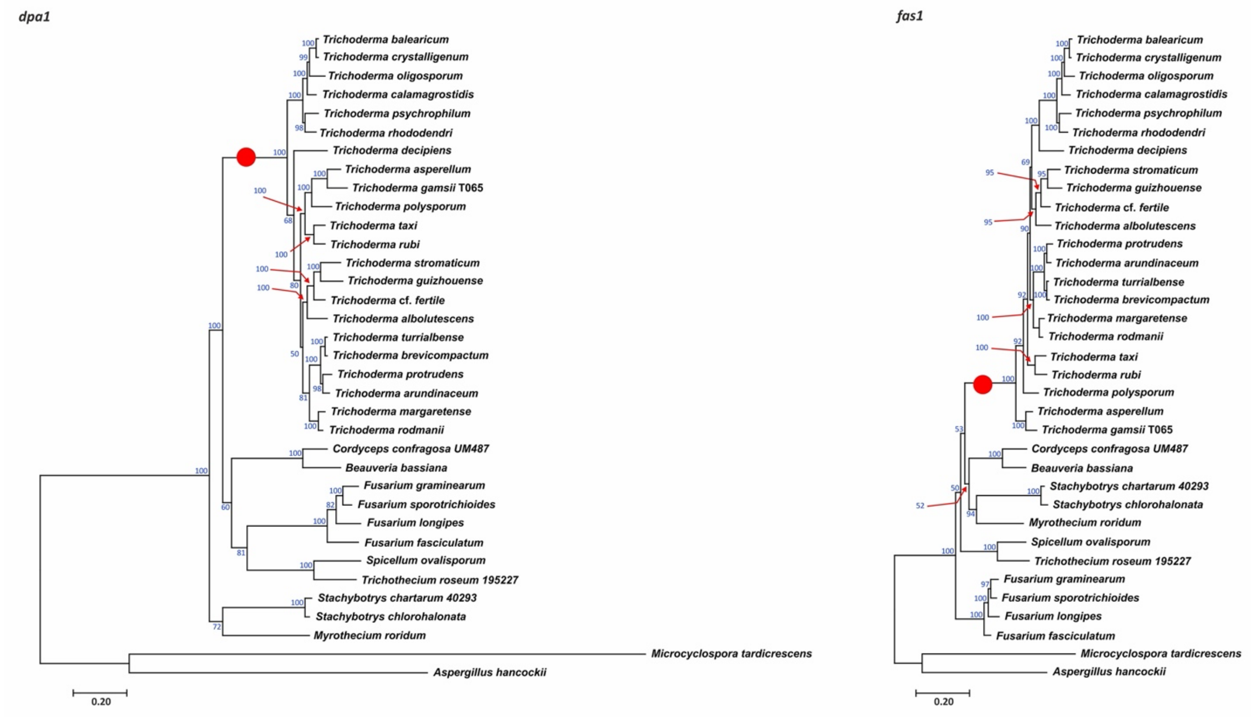

Figure S4. (Cont.)

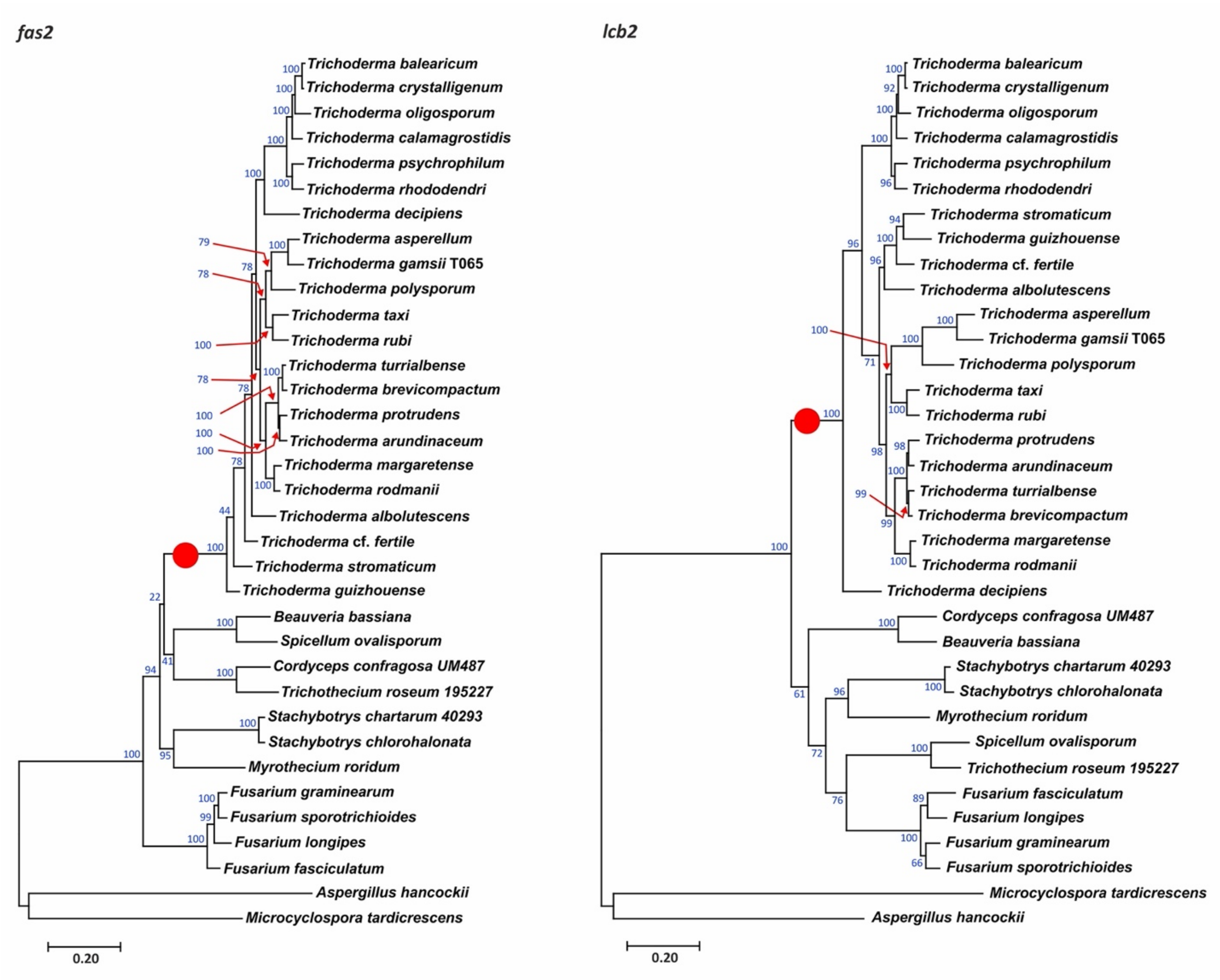

Figure S4. (Cont.)

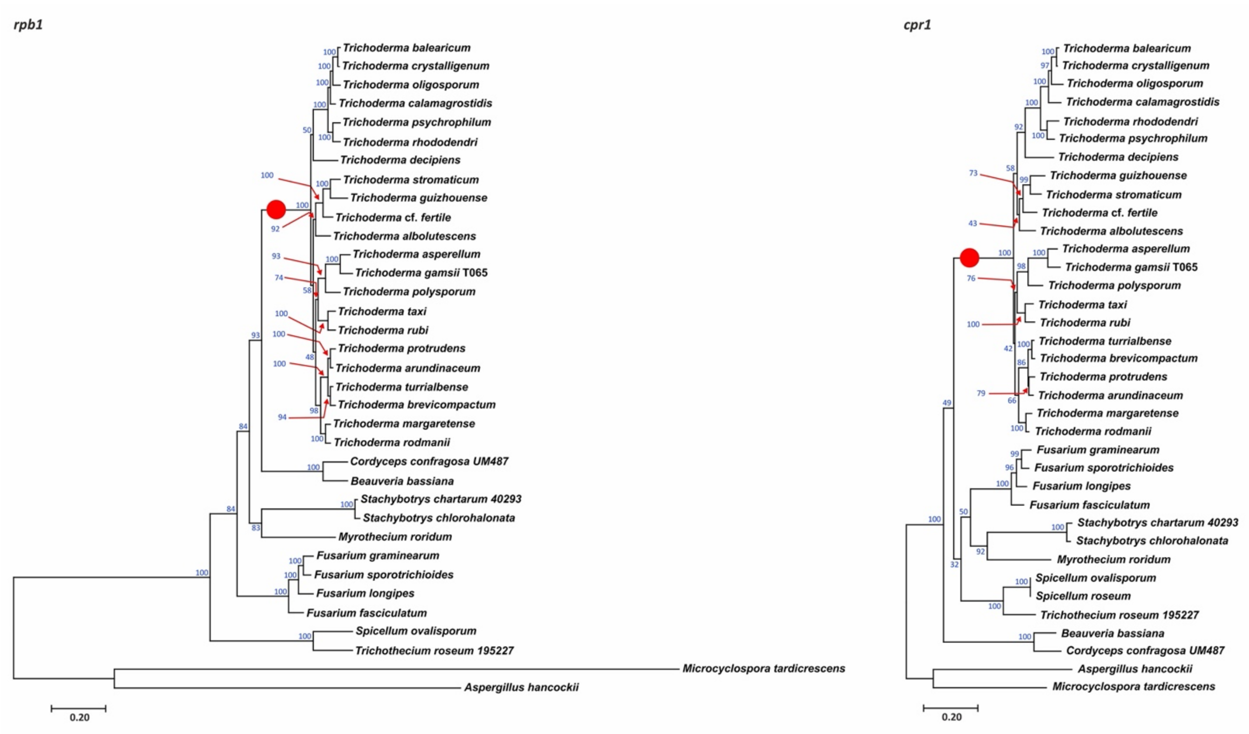

Figure S4. Individual ML Phylogenetic tree of the six genes (*cpr1, dpa1, fas1, fas2, lcb2,* and *rpb1*) used for *tri5* phylogenetic analysis, including all *Trichoderma* species studied in this work and other *tri5* gene-containing fungi. For details regarding the procedures used in the generation of these trees see legend to Fig. 4. The red circle denotes the connecting point between *Trichoderma* species and the other non-*Trichoderma* fungi for each of the genes analyzed.

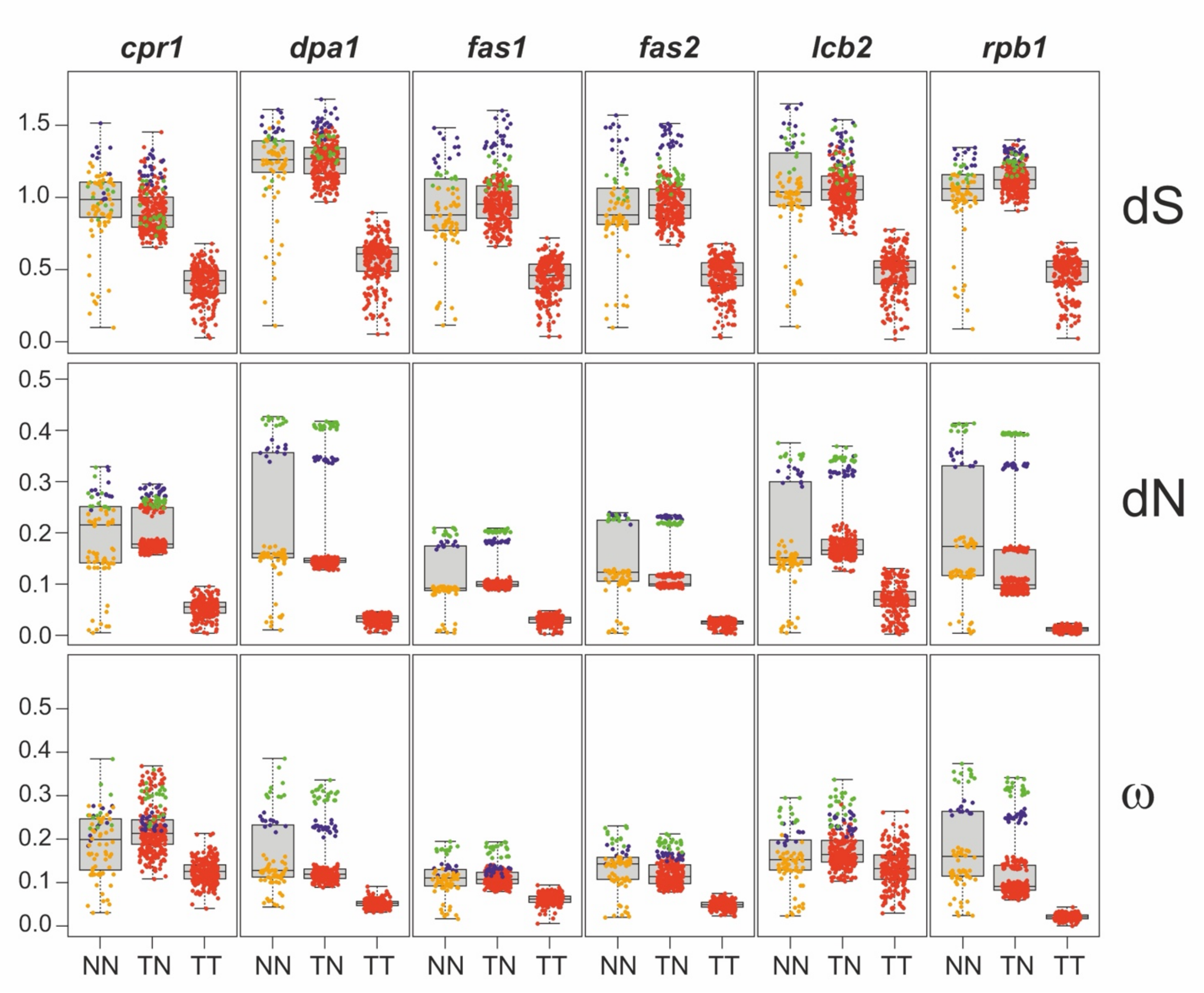

Figure S5. Boxplots and scatterplots of the distances between sequence pairs. The pairs analyzed were between non-*Trichoderma* (NN), *Trichoderma* and non-*Trichoderma* (TN), and *Trichoderma* (TT) genes. The distances of synonymous changes per synonymous site (**dS**); non-synonymous changes per non-synonymous site (**dN**), and their rates (**ω**) are included. Six housekeeping (HK) genes (*cpr1, dpa1, fas1, fas2, lcb2*, and *rpb1*) (Table S2) were considered in this analysis. Green and blue points in the scatterplots represent those that involve *Aspergillus hancockii* or *Microcyclospora tardicrescens* sequences, respectively, while red points correspond to comparisons between all the other sequences used in this study. As it can be observed, the dN, dS, and ω pattern is similar in all HK genes for all comparisons.

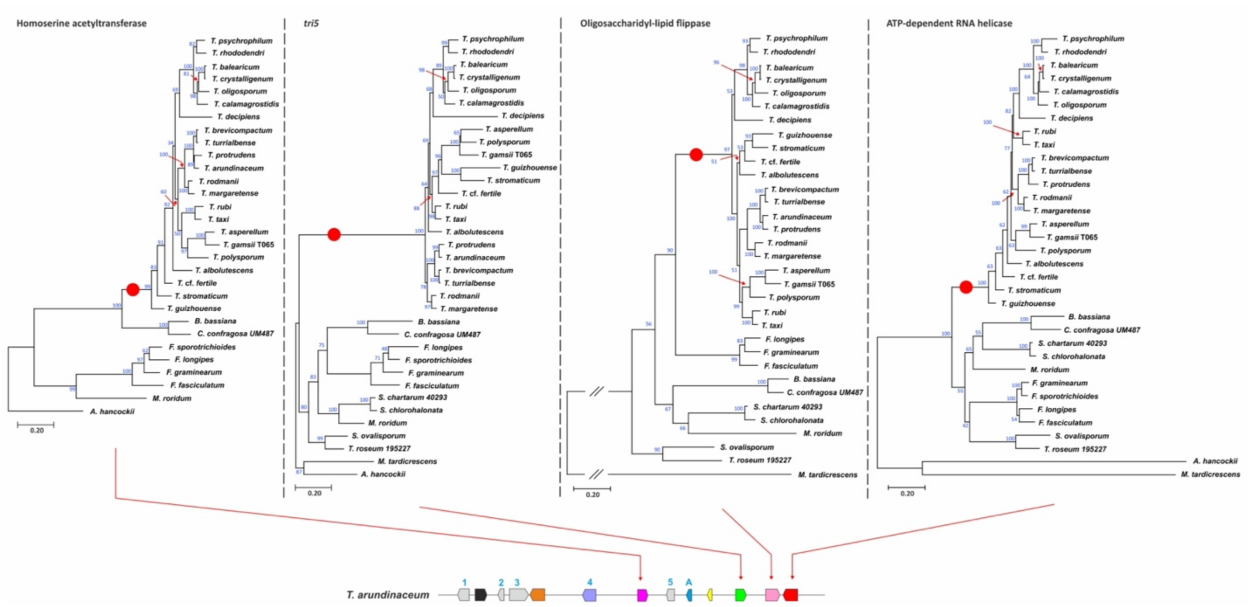

Figure S6. ML Phylogenetic trees of (left to right) the homoserine acetyltransferase, TRI5, oligosaccharidyl-lipid flippase and the ATP-dependent RNA helicase encoding genes located in the *tri5* gene region (see lower panel), including all *Trichoderma* species studied in this work and other *tri5* gene-containing fungi. For details regarding the procedures used in the generation of these trees see legend to Fig. 4. *tri5* tree has been included in the present Figure only for comparative purposes. Note the long *Trichoderma* branch in the *tri5* tree compared to the other trees.

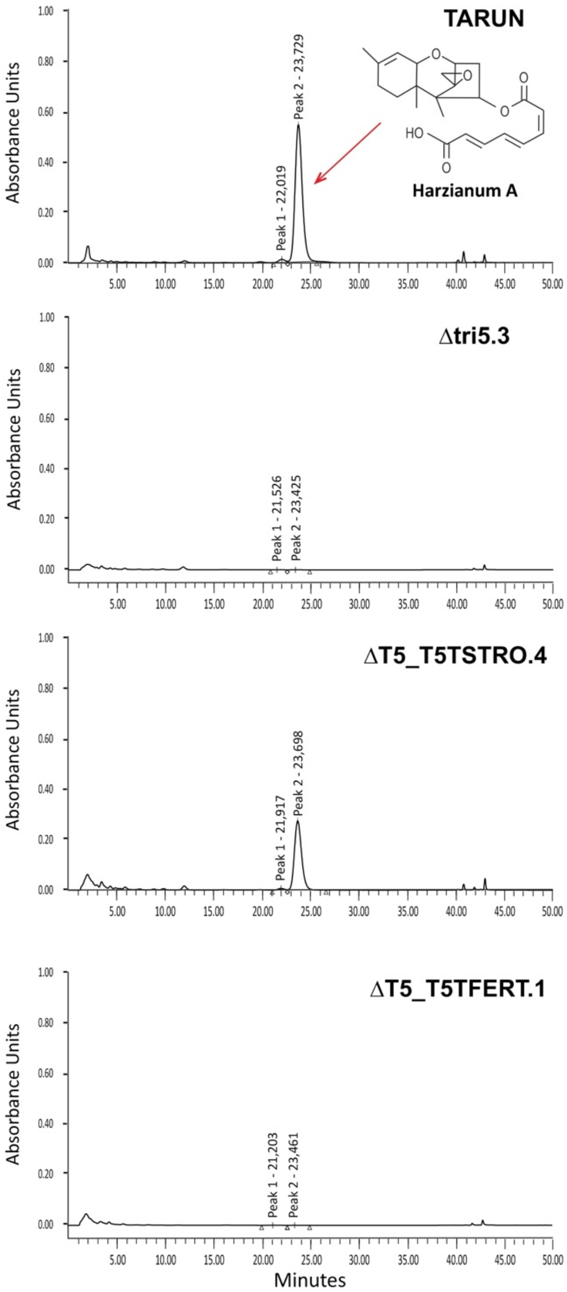

Figure S7. HPLC chromatograms showing production of harzianum A (HA) by *T. arundinaceum* (TARUN) and ΔT5_T5TSTRO.4, a Δtri5.3 complemented transformant with the plasmid overexpressing *T. stromaticum tri5* gene, and the lack of HA production in Δtri5.3 mutant, and in ΔT5_T5TFERT.1, a transformant of Δtri5.3 with plasmid overexpressing *T.* cf. *fertile tri5*.

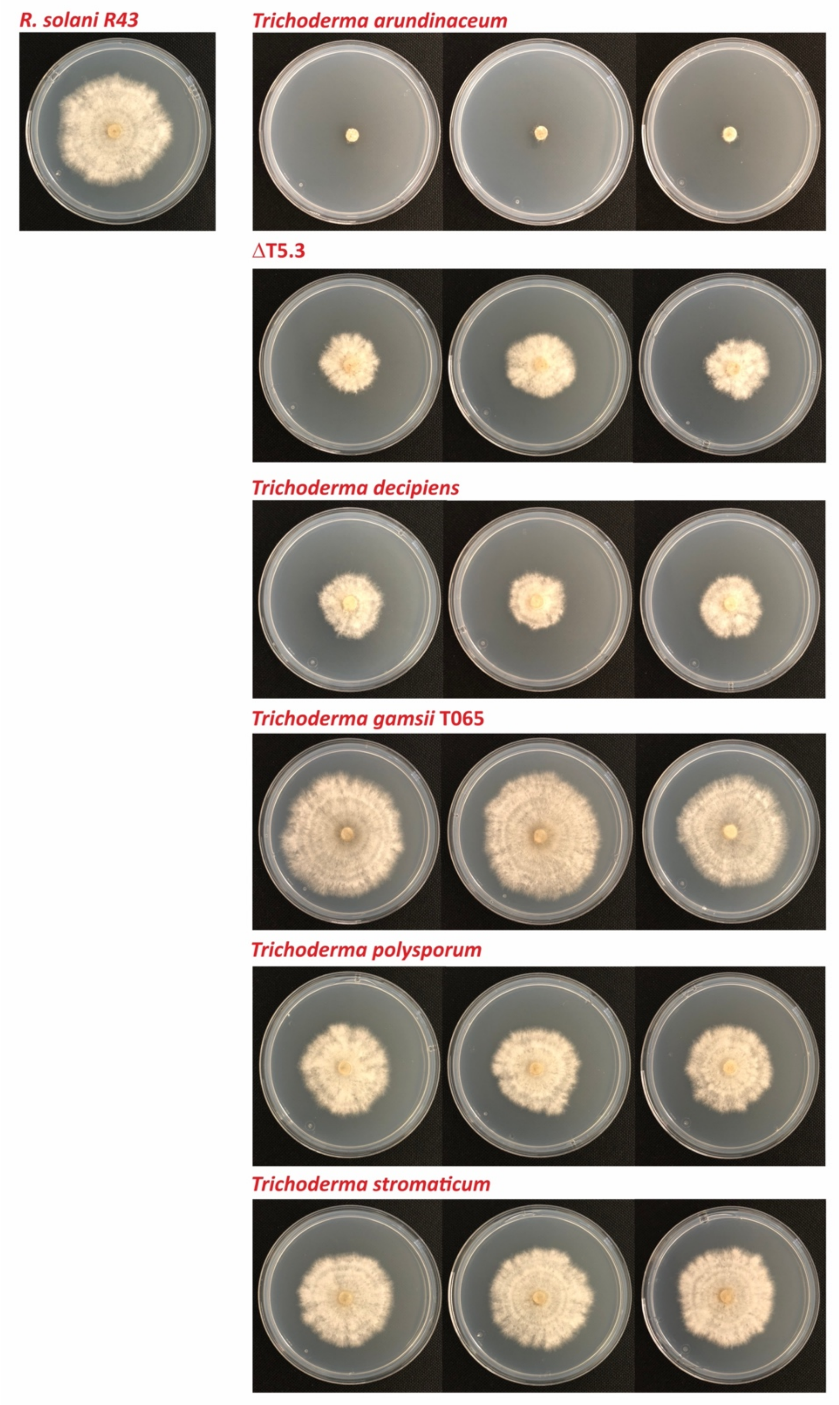

Figure S8. Cellophane membrane assays to analyze the antifungal activity against the fungal phytopathogen *Rhizoctonia solani* R43 of *T. arundinaceum* wild type (TARUN), mutant Δtri5.3 (ΔT5.3), *T. decipiens, T. gamsii* T065*, T. polysporum* and *T. stromaticum.* Note: In this experiment plates were incubated for 5 days after the placement of the pathogen plug.

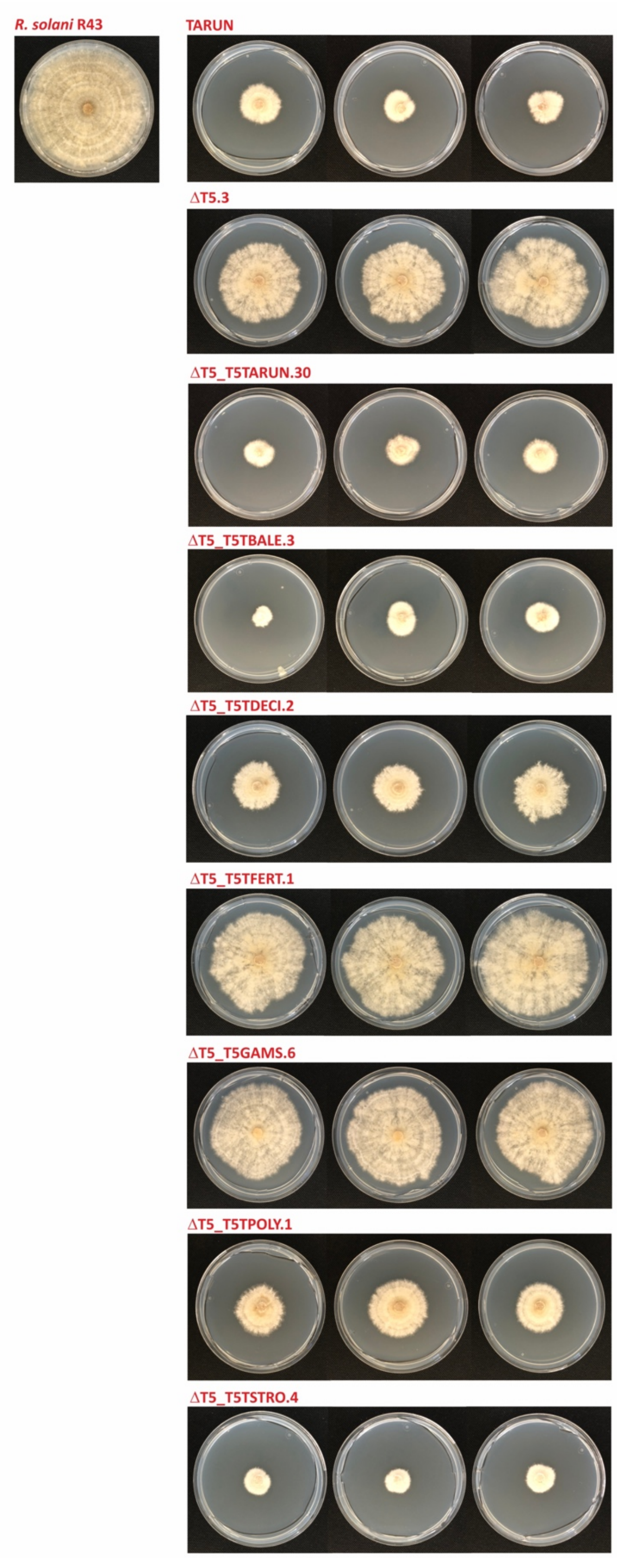

Figure S9. Cellophane membrane assays to analyze the antifungal activity against the fungal phytopathogen *Rhizoctonia solani* R43 of *T. arundinaceum* wild-type (TARUN), mutant Δtri5.3 (ΔT5.3) and transformants of the latter with plasmids overexpressing *tri5* gene from *T. arundinaceum* (ΔT5_T5TARUN#), *T. balearicum* (ΔT5_T5TBALE.#), *T. decipiens* (ΔT5_T5TDECI.#), *T.* cf. *fertile* (ΔT5_T5FERT.#), *T. gamsii* T065 (ΔT5_T5TGAMS.#), *T. polysporum* (ΔT5_T5TPOLY.#), and *T. stromaticum* (ΔT5_T5TSTRO.#). Note: In this experiment plates were incubated for 8 days after the placement of the pathogen plug.

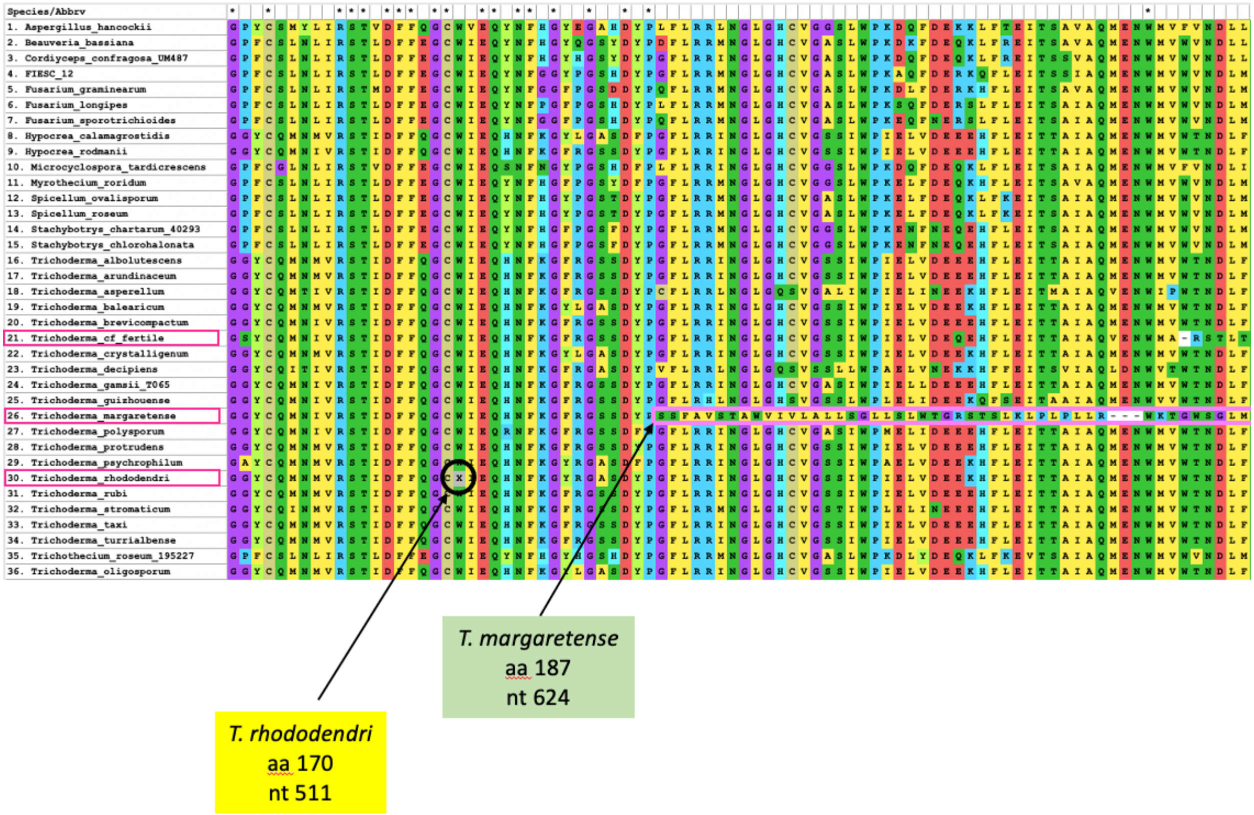

Figure S10a. Image showing frameshifts and non-sense mutations observed in *T. rhododendri* (aa 170) and *T. margaretense* (aa 187) TRI5 proteins.

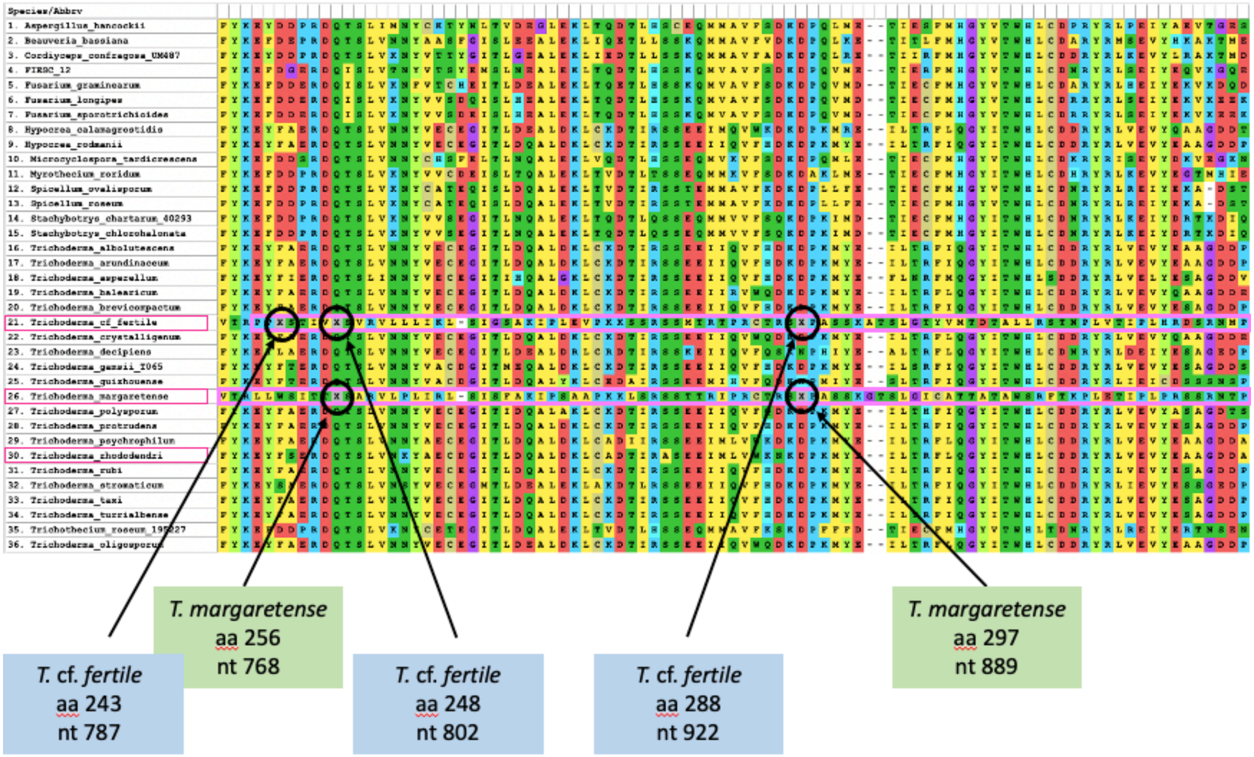

Figure S10b. Frameshift and non-sense mutations located in *T. margaretense* (aa 256, 297) and *T.* cf. *fertile* (aa 243, 248, and 288) TRI5 proteins. FIESC_12= *Fusarium fasciculatum*; *Hypocrea rodmanii= Trichoderma rodmanii; Hypocrea calamagrostidis= Trichoderma calamagrostidis*
